# Green synthesis of trimetallic CuO/Ag/ZnO nanocomposite using *Ziziphus spina-christi* plant extract: characterization, statistically experimental designs, and antimicrobial assessment

**DOI:** 10.1038/s41598-024-67579-5

**Published:** 2024-08-24

**Authors:** Ayman K. El-Sawaf, Shahira H. El-Moslamy, Elbadawy A. Kamoun, Kaizar Hossain

**Affiliations:** 1 Department of Chemistry College of Science and Humanities in Al-Kharj, Prince Sattam Bin Abdulaziz University, 11942 Al-Kharj, Saudi Arabia; 2 Department of Chemistry, Faculty of Science, Menoufia University, Shebin El-Kom, Egypt; 3 Department of Bioprocess Development, Genetic Engineering and Biotechnology Research Institute (GEBRI), City of Scientific Research and Technological Applications (SRTA-City), New Borg Al-Arab City 21934, Alexandria, Egypt; 4 Polymeric Materials Research Department, Advance Technology and New Materials Research Institute (ATNMRI), City of Scientific Research and Technological Applications (SRTA-City), New Borg Al-Arab City, Alexandria, 21934 Egypt; 5 Department of Environmental Science, Asutosh College, University of Calcutta, 92 Shyama Prasad Mukherjee Rd, Jatin Das Park, Bhowanipore, Kolkata, W.B. India

**Keywords:** Ziziphus spina christi leaves, Trimetallic nanocomposite, Green-synthesized ZnO, Antimicrobial activities, Microbiology, Environmental sciences, Chemistry, Materials science, Nanoscience and technology

## Abstract

In this study, *Ziziphus spina christi* leaves was used to synthesize a trimetallic CuO/Ag/ZnO nanocomposite by a simple and green method. Many characterizations e.g. FTIR, UV–vis DRS, SEM–EDX, TEM, XRD, zeta-size analysis, and DLS, were used to confirm green-synthesized trimetallic CuO/Ag/ZnO nanocomposite. The green, synthesized trimetallic CuO/Ag/ZnO nanocomposite exhibited a spherical dot-like structure, with an average particle size of around 7.11 ± 0.67 nm and a zeta potential of 21.5 mV. An extremely homogeneous distribution of signals, including O (79.25%), Cu (13.78%), Zn (4.42%), and Ag (2.55%), is evident on the surface of green-synthetic nanocomposite, according to EDX data. To the best of our knowledge, this is the first study to effectively use an industrially produced green trimetallic CuO/Ag/ZnO nanocomposite as a potent antimicrobial agent by employing different statistically experimental designs. The highest yield of green synthetic trimetallic CuO/Ag/ZnO nanocomposite was (1.65 mg/mL), which was enhanced by 1.85 and 5.7 times; respectively, by using the *Taguchi* approach in comparison to the *Plackett–Burman* strategy and basal condition. A variety of assays techniques were utilized to evaluate the antimicrobial capabilities of the green-synthesized trimetallic CuO/Ag/ZnO nanocomposite at a 200 µg/mL concentration against multidrug-resistant human pathogens. After a 36-h period, the tested 200 µg/mL of the green-synthetic trimetallic CuO/Ag/ZnO nanocomposite effectively reduced the planktonic viable counts of the studied bacteria, *Escherichia coli* and *Staphylococcus aureus*, which showed the highest percentage of biofilm reduction (98.06 ± 0.93 and 97.47 ± 0.65%; respectively).

## Introduction

Nanotechnology is an area of technology that studies, applies, and develops materials at the nanoscopic scale, which typically ranges from 1 to 100 nm^1^. To manufacture nanoparticles, chemical or physical processes are commonly employed. Nevertheless, both processes are challenging to scale up and require large amounts of energy, in addition to potentially dangerous substances^2^. These hazardous substances persist at the interface between nanomaterials, affecting the biocompatibility of nanomaterials. Biological biosynthesis is therefore a well-known solution for these widely used approaches^3^. The green synthesis technique has effectively utilized a wide range of candidates, including bacteria, fungi, algae, and plants. Metallic nanomaterials can be produced by reducing metal ions with the help of the bioactive compounds found in the extracts of these candidates. Because of their large surface-to-volume ratio, these nanomaterials are widely used in many different engineering and materials science domains, including medical, optical, biotechnological, microbiological, electronics, and environmental^4^.

Several areas in Egypt are home to the common medicinal plants *Mentha spp., Ziziphus spina-christi,* and *Ocimum basilicum,* which are highly valued for their anti-inflammatory, antioxidant, antibacterial, and anticancer properties^1–3^. *Ziziphus spina-christi*, a member of the *Rhamnaceae* family, is commonly referred to as Sidr. Together with a number of recently introduced exotic plants, it is an important cultivated tree and one of the few surviving natural tree species in Arabia. The genus *Ziziphus* is well-known for its therapeutic uses as an immune system booster, hypotensive, anti-inflammatory, antimicrobial, antioxidant, and liver-protective agent. Furthermore, there have been reports that the *Z. spina-christi* extract protects against aflatoxicosis. *Mentha spp.,* also known as mint (genus *Mentha*, family *Lamiaceae*), is widely used as a spice component for many different types of food worldwide and is also regularly used to make herbal tea. Because the essential oils of mint include antibacterial and antioxidant qualities, it is widely known that the leaves of the plant are still employed in traditional medicine to treat digestive problems. According to phytochemical analyses of *mentha* plants, the main components of the leaf extracts that reduced and stabilized nanoparticles were phenolic, flavonoid, steroid, and terpenoid^8–10^. The herb sweet basil (*Ocimum basilicum*) has small, pink-tinged, or white blooms and elliptic, bright green leaves, it also has a strong scent.

Many bioactive molecules, including flavonoids, alkaloids, phenolic compounds, sterols, saponins, tannins, and fatty acids, are rich in different plant-based extracts^11^. In the green synthesis of nanoparticles, these molecules act as reductants, capping agents, and stabilizing agents, keeping the resultant nanoparticles stable and preventing them from aggregating completely^12^. According to a previously published study, the crude extract of Ziziphus-Spina Christi leaves (Sider) was utilized in the green production of zinc oxide nanoparticles (38.177 nm) at the hexagonal wurtzite phase^6^. Graphene oxide was phyto-reduced in the other study employing various doses of *Ziziphus spina-christi* aqueous extract^13^. Furthermore, other prior investigations reported a fast and safe synthesis of selenium-doped zinc oxide nanoparticles (50 nm) in spherical shape utilizing aqueous leaf extract (*Mangifera indica*), which showed strong antimicrobial properties^14^. The green synthesis of a safe, stable, and trimetallic nanocomposite containing Cu, Ag, and Zn was achieved previously using an aqueous leaf extract of *Catharanthus roseus*^15^. Previously, *Ocimum basilicum L.* seed extract was used to generate an Ag-doped ZnO-MgO-CaO nanocomposite in a manner that is environmentally friendly^16^. Furthermore, MgO and CuO/MgO nanoparticles were produced via a green approach using an extract from the *Opuntia monacantha* plant^17^. Moreover, a content-based extract from the plant powder (*Ocimum basilicum*) was used in a green method for the biological synthesis of MnO_2_ nanoparticles and MnO_2_@eggshell nanocomposite^18^. Also, *Calotropis gigantea* leaf extract was used to generate the green-manufactured binary ZnO-CuO nanocomposites, which show promising antimicrobial properties against skin-related infections^11^. Meanwhile, silver nanoparticles (Ag NPs) that are cost-effective and environmentally friendly were produced using the aqueous extract of *Ziziphus spina-christi* leaves for treating Fusarium wilt disease^19^. Besides, Poly(HEMA-co-FAOEME)/ZnO nanocomposites were generated as an antimicrobial agent by biosynthesizing ZnO nanoparticles using *Mentha plegium L.* extract^20^. ZnO, MgO, CuO, and their composite mixed oxide nanoparticles were previously manufactured utilizing a green approach and leaf extracts of medicinal plants e.g.* Pisonia grandis R.Br.*^21^. The antimicrobial and pro-healing abilities of silver, copper, and zinc oxide nanoparticles are widely recognized. Because of their photo-oxidizing and photo-catalytic effects on biological species, these nanoparticles are safe and biocompatible nanomaterials^22^.

To prove their antimicrobial properties, these nanoparticles may interact chemically as well as physically. As a result of these nanomaterials' interactions with microbial cells, reactive oxygen species (ROS), H_2_O_2_, and ions are released under photoinduced conditions^23^. Conversely, depending on the examined nanomaterials, physical interaction may exhibit biocidal impacts through cellular internalization, breakdown of the cell membrane, or forceful damage^24^. Furthermore, it has been suggested that ZnO-Ag NCs have the strongest capacity to break down microbial cell membranes and interact with vital DNA elements such as phosphorus and sulfur, inhibiting DNA replication^23,25,26^. The higher specific surface area to volume ratio of the nanoparticles led to the formation of more ROS, which is dependent on binding and interacting with the cell membrane and aggregating in the lipid layer^23^. The negative charge of super oxides and hydroxide ions allows them to enter microbial cells. Eventually, this may result in the cell wall breaking down, releasing its contents, and finally causing cell death^27^. Damaged electrostatic interactions lead to the mortality of pathogens when the negative charge on the surface of the cell membrane catches the positive charge on emitted ions from nanomaterials^26,28^. Our results can be explained by the green, synthesized trimetallic CuO/Ag/ZnO nanocomposite's high infusibility and ability to generate more released ions e.g. (Ag^+1^, Zn^+2^, and Cu^+2^ ions)^23^. Additionally, the released ions penetrated the host cell by binding to surface proteins on the cell wall. Afterward, the microbe's cells died as a result of the altered metabolism^29^.

As environmental problems throughout the world become more urgent, scientists are looking into the possibility of using nanomaterials to address these problems. Scientists have recently focused their attention on nanomaterials and nanocomposites developed from plant extracts. Our group was drawn to the trend of using plant extracts to produce nanocomposites on a large scale. When compared to alternative fabrication methods, biosynthesized nanoparticles are less costly, non-toxic, and very stable. Biomolecules produced by plants are extensive and can be used to generate nanomaterials for a variety of biological applications.

To the best of our knowledge, no previous reports of a trimetallic CuO/Ag/ZnO nanocomposite that was produced environmentally employing a *Ziziphus spina christi* leaf extract, have been published to date. Thus, different experimental designs, such as the *Plackett–Burman* and *Genichi Taguchi* procedures, were also targeted in this work to commercially optimize green-synthesized trimetallic CuO/Ag/ZnO nanocomposite as a strong antimicrobial ingredient.

## Materials and methods

### Materials

A variety of human pathogens, such as *Escherichia coli* (ATCC 10536), *Klebsiella pneumoniae* (ATCC 10031), *Staphylococcus aureus* (ATCC 25923), *Bacillus subtilis* (ATCC 11774), *Candida albicans* (ATCC 10231), and *Candida krusei* (ATCC 6258), were used to assess the antimicrobial efficacy of the green synesthetic nanocomposites. All human pathogens were received from GEBRI, SRTA-City, Alexandria, Egypt. Fresh leaves of *Mentha, Ocimum basilicum,* and *Ziziphus spina hristi* were collected from the New Borg Al-Arab City farms in Alexandria, Egypt.

### Preparation of *Ziziphus* spina-christi plant extract

Three well-known herbal plants i.e. (*Mentha, Ziziphus spina-christi),* and *(Ocimum basilicum)* were gathered locally for this study. Greenish-yellow leaves were collected from these plants and washed twice under running water before being thoroughly cleansed with distilled water to remove any extra remaining dirt. After carefully cleaning every leaf with a white cloth, the leaves were allowed to air dry for three hours. It was ground into a fine powder after dried for 72 h at 60 °C. Finally, 10 g of dry powder and 100 mL of double-distilled water were added to a 250-mL *Erlenmeyer* flask and shaken at 200 rpm at 70 °C for 30 min to extract the components. To eliminate any last bits of tiny plant debris from the recovered material, centrifugation at 6000 rpm revolutions per minute was performed using Whatman No. 1 filter paper. The filtered extract was stored at 4 °C for future experimental use^16,30,31^.

### Quantification of main leaf extract constituents

With a minor modification, the *Folin-Ciocalteu* method was utilized to determine the total phenolic content^32^. In brief, 40 μL of plant extract was mixed with 1.8 mL of 2N Folin-Ciocalteu for 5 min at room temperature (25 °C). The resulting mixture was subsequently mixed with 1.2 mL of a 7.5% sodium carbonate solution and allowed to react for one hour in the dark at ambient temperature, where the absorbance at 765 nm was finally measured. The standard component was gallic acid (y = 0.6812x + 0.0314, R^2^ = 0.9975), and the sample’s total phenolic content was expressed in milligrams of gallic acid equivalents (mg GAE/g). Rutin, a slightly modified standard substance, was utilized with the aluminum chloride method to evaluate the total flavonoid concentration^33^. Overall, 1 mL of extracts and blank (H_2_O) were mixed with 3 mL of potassium acetate (0.1 mol/L) and 2 mL of aluminum chloride solution (0.1 mol/L). After allowing the mixture to react for 20 min, 70% aqueous ethanol (v/v) was added to dilute it to a final volume of 10 mL. The standard curve was (y = 1.4715x + 0.0364) (R^2^ = 0.9998). The absorbance was finally measured at 510 nm, and the results were reported as rutin equivalents (mg RE/g). The total protein content was calculated using *Bradford’s* (1976) method and expressed as mg/g fresh weight (FW). To prepare one gram of fresh plant tissues for protein and enzyme extractions, three milliliters of 25 mM Tris–HCl buffer (pH 6.8) and 3% polyvinylpolypyrrolidone were homogenized at 4 °C. Protein analysis was completed using the supernatant after the resulting mixture was centrifuged for an hour at 13,000 rpm at 4 °C. The concentrations in mg/g FW were determined using standard curves for each reducing sugar^34^. The extract was centrifuged at 12,000 rpm, and the supernatant was kept in the dark for a whole day to determine anthocyanins^35^. After that, an absorbance measurement at 550 nm was taken for all the samples. After computing the total anthocyanin content using a coefficient of attrition of 33,000 mM/cm, the result was expressed as µg/g final weight.

### Green synthesis of trimetallic CuO/Ag/ZnO nanocomposite

A 250-mL Erlenmeyer flask was filled with 50 mL of each diluted plant extract (50%) and was agitated for 30 min, while 0.1M AgNO_3_, 0.1M Cu (NO_3_)_2_.3H_2_O, and 0.1M Zn (CH_3_COO)_2_.2H_2_O were titrated gradually at a time. The solution was then continually stirred at 80 °C, while the pH was adjusted to (5.5, 7.0, 14) using 2M NaOH solution. A precipitate that was dark brown developed after this reaction, was stirred for two hours. The precipitate that was produced was centrifuged for 15 min at 12,000 rpm, and after being repeatedly cleaned to remove contaminants with distilled water and ethanol, the pelt was dried for two hours at 80°C. A mortar and pestle were used to grind the dried pelt into a powder, and dry weights were estimated for each plant extract. UV–visible spectroscopy (Shimadzu, Japan) was also used to identify the absorbance bands and band gaps to confirm that the nanocomposite was synthesized utilizing each of the extracted plants.

### Bioassay survey

An agar-well diffusion method was used to determine the green synthesized nanocomposite’s antimicrobial sensitivity in vitro, against a range of multi-drug-resistant human pathogens. The investigated pathogens were cultured in individual culture inoculated in sterile nutrient broth containing (0.5% peptone, 0.5% NaCl, and 0.3% yeast extract). The inocula was then incubated for 24 h at 37 °C. After incubation, the individual culture suspension was utilized for the bioassay survey. A sterile well cutter was used to cut 5-mm-diameter wells on Muller Hinton agar medium (0.2% beef extract, 0.15% starch, 1.75% casein, and 1.7% agar). Subsequently, 0.1 mL of every pathogen was spread out on the agar plates, and 50 μL of the nanocomposites that synthesized at distinct pH levels (pH 5, 7.0, and 14) were added to the hollows. 20 μL of *Ziziphus spina-christi* extract was used as a control. The inoculation culture plates were held at 4 °C for 5 h before incubation for 48 h at 37 °C. After incubation, the inhibitory zones that formed were measured in millimeters.

### Characterization of green synthesized nanocomposite

FTIR spectra were investigated using a JASCO-410 spectrometer (JASCO, Easton, MD). To further explore the surface morphology of the green synthetic nanocomposite, a SEM (Qattro, Thermo-Scientific, USA) JSM-6510LV, USA was utilized. The transmission electron microscope was additionally utilized for analyzing the nano structural features (TEM, JEM-2100F, JEOL: Japan). Thermal stability of synthesized nanocomposites was assessed using (TGA, DTA, and DSC) was estimated at 29–1000 °C utilizing a DSC-TGA device model (SDTQ 600, USA) under a N2 atmosphere (flow rate of 100 mL/min and a heating rate of 10°C/min). Furthermore, (Horiba, SZ-100, Kyoto, Japan) specimen was used to investigate the green synthetic nanocomposite’s zeta potential using dynamic light scattering (DLS). A temperature of 25 °C was maintained during a 20-min dilution and dispersion process in an ultrasonic bath for examining the green synthetic nanocomposite in a DLS machine.

### Statistical optimization to maximize the yield of green-synthesized nanocomposite

Two successive experimental designs were used in this work to maximize the yield of nanocomposite’s green-synthetic reaction. The factors affecting the green synesthetic reaction were assessed using the *Plackett–Burman* and *Taguchi* designs, such as concentrations of plant extract (F1), concentrations of precursors (F2), ratio of precursors (F3), reaction agitation (F4), reaction temperature (F5), reaction pH (F6), and incubation period (F7).

#### Plackett–Burman design (PBD)

In several investigations, this design was utilized to evaluate the rate of green synesthetic reaction and the overall yield of dry-weight nanocomposite^36–38^. Green-synthetic reaction variables are used in these qualitative and quantitative screening procedures to identify the ideal parameters for maximizing the dry weight of nanocomposite products. The yield weight of green synthetic trimetallic CuO/Ag/ZnO nanocomposite was found to be affected by seven factors, which included concentrations of plant extract, concentrations of precursors, ratios of precursors, reaction agitation, temperature, reaction pH, and incubation time. These factors were selected based on previous experiments (data not shown). As indicated in Table 1, these factors were examined at two different levels: the highest (1) and the lowest (− 1).

**Table 1:** Factors employed in *Plackett–Burman* and their corresponding values for optimizing the yield of green synesthetic nanocomposite.

Factors	Codes	Units	Low (− 1)	High (+ 1)
Concentrations of plant extract^a^	F1	%	10	100
Concentrations of precursors^b^	F2	M	0.5	2
Ratio of precursors^c^	F3	v/v/v	1:01:01	1:01:04
Reaction agitation	F4	RPM	10	250
Reaction temperature	F5	°C	80	100
Reaction pH	F6	–	4	8
Incubation time	F7	h	0	6

^a^*Ziziphus spina-christi* is the plant extract that was tested.

^b^AgNO_3_, Cu (NO_3_)_2_.3H_2_O, and Zn (CH_3_COO)_2_.2H_2_O were the used precursors.

^c^Ratio of the tested precursors (AgNO_3_: Cu (NO_3_)_2_.3H_2_O: Zn (CH_3_COO)_2_.2H_2_O).

The response was calculated using the average green synthesized nanocomposite dry weight, and each experiment was conducted twice. A first-order polynomial model serves as the foundation for mathematical modeling of PBD, as shown in Eq. (1). In this case, Y is the dry weight of nanocomposite that were biosynthesized (response), β_0_ denotes the model intercept, β_i_ is the linear coefficient, and X_i_ is the number of independent variables.

1 $$ Y \left( {\text{dry weight of nanocomposite }} \right) = \beta_{0} + \sum \beta_{i} X_{i} $$

Furthermore, Eq. (2) was used to calculate the efficiency of each variable. In this equation, M_v_ represents the variable main effect, Mv + and Mv− are the cell dry weights in trials where the independent variable was present at high and low levels; respectively, and N is the number of trials divided by two.

2 $$ M_{v} = \frac{{\left( {\sum M_{v + } - \sum M_{v - } } \right)}}{N} $$

*Minitab® 18.1* software was utilized to generate a set of 12 trails for statistical analysis and graph charting. All independent variables were evaluated for their impact on the response using analysis of variance (ANOVA), with a significance level of P < 0.05. The fitness of the equation was assessed using the multiple correlation coefficient (R^2^) and adjusted R^2^.

*Taguchi* technique: To generate a valid result, the *Taguchi* technique was built up in several steps: choosing important components, creating an accurate matrix, analyzing statistical data, and finally validating using the best values. The goal of this work is to use several criteria to determine the maximum cell dry weight of the overall dry-weight nanocomposite yield (Table 2). The L27(3^7) *Taguchi* orthogonal array design was used for this optimization technique (7 factors, 3 levels, and 27 runs). An orthogonal array (signal-to-noise ratio, or "S/N") is created by first identifying the factor levels (inner array) using numbers like 1, 2, 3 etc. These levels are then compared to different combinations of noise factors in the outer array. The S/N ratio is expressed in decibels (dB).

**Table 2:** Factors employed in the *Taguchi design* and the associated probabilities for maximizing the production efficiency of green synesthetic nanocomposites.

Factors	Codes	Units	Levels of parameters		
			1	2	3
Concentrations of plant extract^a^	F1	%	25	50	75
Concentrations of precursors^b^	F2	M	0.25	0.5	0.75
Ratio of precursors^c^	F3	v/v/v	1:1:1	2:1:1	1:2:1
Reaction agitation	F4	RPM	50	150	200
Reaction temperature	F5	°C	30	40	50
Reaction pH	F6	–	5	6	7
Incubation time	F7	h	1	2	3

^a^Ziziphus spina-christi is the plant extract that was tested.

^b^AgNO_3_, Cu(NO_3_)_2_.3H_2_O, and Zn (CH_3_COO)_2_.2H_2_O were the used precursors.

^c^Ratio of the tested precursors (AgNO_3_: Cu (NO_3_)_2_.3H_2_O: Zn (CH_3_COO)_2_.2H_2_O).

Once the average of produced cell dry weights and the signal-to-noise (S/N) ratio (the larger the better group) are determined for each process condition as designed, the F test and *ANOVA* are used to examine the significance of all factors and their relationships at levels using the *MINITAB 18* software. At the end of the process, a confirmation test was conducted to compare the experimental value with the results that were achieved using Taguchi's method. The S/N ratio is expressed in decibels [dB] and calculated using Eq. (3), where n is the number of observations and Y is the observed data (dry-weight nanocomposite yield).

3 $$ \frac{S}{N}ratio \left[ {dB} \right] = - 10 {\text{Log}}\left[ \frac{1}{n} \right] \mathop \sum \limits_{i = 1}^{n} \frac{1}{{Y_{i}^{2} }} $$

Moreover, the predicted S/N ratio was estimated, where n is the number of parameters, S/Nm is the total mean S/N ratio, and S/Ni is the mean S/N ratio at the optimum level using Eq. (4).

4 $$ Predicted \left( \frac{S}{N} \right)ratio \left[ {dB} \right] = \frac{S}{{N_{m} }} + \mathop \sum \limits_{i = 1}^{n} \left[ {\frac{S}{{N_{i} }} - \frac{S}{{N_{m} }}} \right] $$

### Antimicrobial efficiency bioassays

The CLSI standard^39^, was followed in the agar well-diffusion method, biofilm inhibition assay, and time-kill experiment employed to evaluate the antimicrobial efficiency of green synthesized CuO/Ag/ZnO nanocomposite.

#### Agar well-diffusion technique

The studied human pathogens were cultured in a nutrient broth medium (0.5% peptone, 0.3% yeast extract, 0.2% beef extract, and 0.5 NaCl) to achieve the 0.5 McFarland turbidity standards. On nutrient agar plates, 100 µL of microbial cultures were spread out using sterile cotton swabs. For this experiment, three different dosages of the selected green synesthetic nanocomposite designated as (A): 50 µg/mL, (B): 100 µg/mL, and (C): 150 µg/mL were prepared. Immediately after using a sterile 6-mm cork-borer to drill each well, 50 µL of the evaluated green synesthetic nanocomposite dosage was added. After that, the agar plates were incubated for 24 h at 37°C. A ruler was used to measure the inhibitory zone, or the clean zone, in millimeters (mm) around each well^40^.

#### Biofilm inhibition assay

The broth microdilution method was used to estimate the minimum inhibitory concentration of the examined green synesthetic nanocomposite by calculating the lowest concentration at which no discernible growth appeared^41^. To measure MIC, multiple dosages of the selected green synesthetic nanocomposite, ranging from 50 to 250 µg/mL were generated. The tested pathogens were cultured individually in nutrient broth medium at 37 °C and 150 rpm to generate the pre-inoculums. To obtain (2 × 10^5^) CFU/mL, each pathogen was separately inoculated into a fresh nutrient broth, which was then incubated at 37 °C and 150 rpm. The optical density (OD) at 600 nm was measured over a 6-h incubation period to determine a microbe's exponential phase. Aseptically, 100 µL of each dosage of the tested green nanocomposite was put into 900 µL of these planktonic cultures to generate treated cultures. Moreover, green synesthetic nanocomposite-free cultures were employed for developing untreated (control) cultures. The microbiological turbidity was measured spectrophotometrically to evaluate the inhibitory effects of the tested chemical compounds. The percentage of anti-biofilm in each sample was determined by using Eq. (5) to compare OD of the treated culture (T) to the corresponding untreated culture (U).

5 $$Reduction in generated biofilm (\%)=\left[\frac{\left(U-T\right)}{U}\right]\times 100$$

#### Time kill-kinetics assay

Macro-broth dilution method was employed, to reach the early logarithmic stage; every one of such pathogens was inoculated separately into nutrient broth medium and incubated for 6 h at 37 °C while being agitated at 150 rpm. The inoculum from each microbial culture (5 × 10^8^ CFU/mL) was then transferred to 9 mL of freshly made nutrient broth medium. The green synesthetic nanocomposite (1 mL) was then added at a concentration of 200 µg/mL. A control growth system was prepared for each pathogen, omitting the tested formulation. After that, these tubes were shaken constantly at 150 rpm and 37 °C for the rest of the period of incubation. Under aseptic conditions, the samples were routinely taken at many time intervals (0, 6, 12, 18, 24, 30, 36, 42, and 48 h). After diluting the samples with sterile saline, 100 µL of the mixture was swabbed onto nutrient agar plates. During the incubation periods, the number of visible colonies was counted and reported as CFU/mL. The percentage of the pathogen's cells' biofilm reduction exposed to the tested green synesthetic nanocomposite with each control was determined using the logarithm of the counted colonies (Log_10_CFU/ml) for each time interval (Eq. 6). Furthermore, by determining the lowest dose that eliminated at least 99.9% of the initial microbial cells, the minimum bactericidal concentration (MBC) was determined.

6 $$Biofilm inhibition rate (\%)=\left[\frac{\left({\text{log}}_{10}\frac{CFU}{ml}_{Untreated}-{\text{log}}_{10}\frac{CFU}{ml}_{Treated}\right)}{{\text{log}}_{10}\frac{CFU}{ml}_{Untreated}}\right]\times 100.$$

### Statistical analyses

The results of antimicrobial efficacy tests were performed in triplicate, and the mean ± standard deviation (M ± SD) was utilized to describe the results. Tukey’s multiple comparison post hoc test was utilized in the *Minitab 19* program (*MINITAB version 19.1*) to compute a one-way analysis of variance (*ANOVA*) and confirm statistical significance. A 95% confidence interval was used for statistical significance (p < 0.05).

## Results and discussion

### Biosynthetic of green-synthesized trimetallic nanocomposite

Basically, nanostructures can be synthesized using sol–gel processing, hydrolysis/condensation, and wet chemical processing. These techniques are mostly costly, require exact experimental parameters (temperature, pressure, energy, and timeframe), and require toxic traditional chemicals. However, green synthesis of nanostructures is attracting a lot of interest currently, due to many significant advantages including simpler, cheaper, and more eco-friendly technique. The most promising method of synthesis is "*green synthesis*," which is achieved by employing either plant extracts or specific microbes (bacteria, fungi, algae, etc.). Many studies on the synthesis of various metals nanoparticles including Zn, Mn, Cu, Au, and Ag, which have been carried out in recent years with a focus on different types of biological systems^42^. In pharmaceutical formulations, medicinal plant extracts are utilized for their bioactive ingredients, helping in the reduction and capping of metal ions through the synthesis of nanostructures^43^. For instance, zinc nitrate ionization in an aqueous solution produced Zn^2+^, which was subsequently reduced to Zn^+^ by a phytochemical present in the extract (functional as reducing, capping, and stabilizing agents). Chemicals containing phenolic groups and hydroxyl groups may hydrolyze and generate nanostructures. Among the several kinds of metallic nanoparticles, Ag, CuO, and ZnO have attracted the attention of many scientists, due to their many applications in different scientific sectors^44–46^. These nanoparticles have been extensively employed in antimicrobial, antioxidant, and photocatalytic applications^44^. On the connections between these metals in plant extract-based nanocomposites, however, there is currently no information available. Due to the synergistic effect of their respective qualities, this combination usually improves the material's properties^47^.

Thus, our work contributes to the effort to find a novel material with remarkable physiological characteristics that has been produced using green techniques. Polyphenols (including flavonoids and saponins), alkaloids, proteins, phenolic acids, sugars, and terpenoids all of which are found in various plant parts—help reduce and stabilize metal ions to produce nanostructures^48^. Thus, the use of plant extracts not only saves energy, time, and steps while reducing the use of toxic chemicals, which protects the environment and human health, but also enhances the efficacy and properties of nanoparticles in the pharmaceutical and medical fields by retaining active chemical molecules on their surfaces^49^. The green synthesis of nanocomposites in aqueous plant extracts is suggested to be influenced by a variety of bioactive molecules, including proteins, polyphenols, and polysaccharides^50^. So, the examined plant extracts were evaluated by determining their constituents. A set of methods was used to test specific components of the various leaf materials of *Mentha*, *Ocimum basilicum*, and *Ziziphus spina christi* before the green synthesis of CuO/Ag/ZnO nanocomposites, were developed. Table 3 initially reports the results of the determinations for total protein, reducing sugar, anthocyanin, phenol, and flavonoids. According to phytochemical investigations, the main constituents of the tested leaf extracts that contributed to the stabilization and reduction of nanoparticles were protein content, reducing sugar, flavonoids, phenolics, and anthocyanin. The results showed that these constituents were richest in *Ziziphus spina-christi*, *Ocimum basilicum*, followed by *Mentha spp*. The results showed that the *Ziziphus spina christi* extract consisted of high levels of flavonoids (26.60 ± 2.25), total phenolic compounds (35.69 ± 5.38), reducing sugar (2.84 ± 0.22), anthocyanin (5.92 ± 0.05), and total protein (2.96 ± 0.27).

**Table 3:** Estimated main elements of the tested plant extracts.

Plant extract	Total protein (mg/g FW)	Reducing sugar (mg/g FW)	Anthocyanin (mg/g FW)	Phenol (mg/g FW)	Flavonoids (mg/g FW)
*Mentha spp.*	0.39 ± 0.36	1.98 ± 0.06	0.98 ± 0.87	25.54 ± 2.58	14.22 ± 1.87
*Ziziphus spina-christi*	2.96 ± 0.27	2.84 ± 0.22	5.92 ± 0.05	35.69 ± 5.38	26.60 ± 2.25
*Ocimum basilicum*	1.22 ± 0.09	2.09 ± 0.58	3.68 ± 1.03	29.58 ± 0.07	19.55 ± 2.54

**Figure 1:**
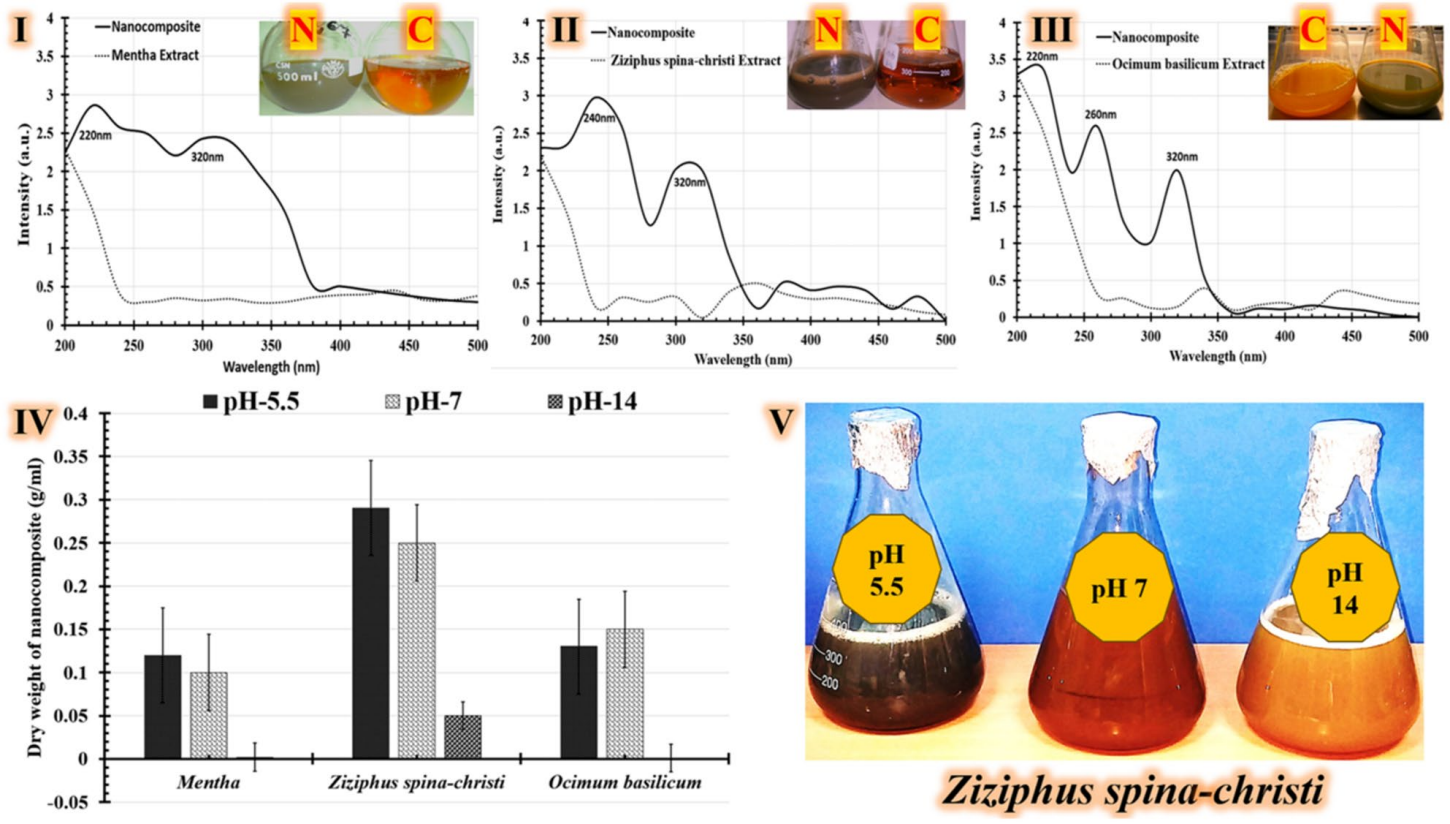
Findings of green synthesized nanocomposite generated from various aromatic plant extracts. UV–vis plots for the prepared nanocomposite, compared to the examined plant extracts: *Mentha spp.* (**I**), *Ziziphus spina-christi* (**II**), and *Ocimum basilicum* (**III**). Photos of the extract plants (**C**) and the yield nanocomposite (**N**). The chart depicts the dry weights of nanocomposites generated from various aromatic plant extracts at different pH levels (**IV**). The color of the resulting green nanocomposite synthesized with *Ziziphus spina-christi* at various pH levels (**V**).

These aromatic plant extracts (reductants) were then titrated under shaking conditions with the precursors composed of (0.1M AgNO_3_, 0.1M Cu(NO_3_)_2_.3H_2_O, and 0.1M Zn(CH_3_COO)_2_.2H_2_O) together to generate a green synesthetic trimetallic nanocomposite. The reaction color changed from reddish yellow (aromatic plant extracts) to dark turbid brown, indicating that the extracts of *Mentha spp.* (Fig. 1IC), *Ziziphus spina-christi* (Fig. 1IIC), and *Ocimum basilicum* (Fig. 1IIIC) generated a green synthetic nanocomposite (Fig. 1I,II, and IIIN). An essential technique for figuring out the electronic structure, optical activities, and physico-chemical characteristics of nanoparticles is UV–visible (UV–vis) spectroscopy^51^. The classification of nanoparticles in the size range of 2–100 nm was found to be adequate for absorption of wavelengths 200–800^52^. The absorption edge of our green synthetic nanocomposite was estimated using a spectrophotometer scanning a range of 200–500 nm, and the results were compared with the extracts employed in each case. The real absorbance was graphed from 0 to 4.0 au. using the *Origin Pro* software (v. 8.0, OriginLab Co., Northampton, MA, USA) to generate fitted curves.

The real green synthetic nanocomposite consistently displays distinct absorption peaks at 220 nm (Fig. 1I), 240 nm (Fig. 1II), and 260 nm (Fig. 1III), in addition to 320 nm when its wavelength is compared to the extract's peaks. A green-generated trimetallic Cu, Zn, and Ag nanocomposite utilizing *Catharanthus roseus* leaf extract has shown similar results elsewhere^53^. According to reports, the absorption bands for Cu, Zn, and Ag nanocomposites have been identified at 220, 270, and 370 nm; respectively^15^. In addition, an experiment revealed the existence of zinc ions in the crystal lattices, which caused the lattices to shift, especially in consideration of their extension. The absorbance peak's strength increases as a result of this alteration after ZnO doping with Cu^54^. Furthermore, a rise in the absorbance band at 220 nm confirms the presence of copper oxide (CuO)^54^. This peak is found in a similar range by numerous other investigations that demonstrated the generation of CuO NPs. Zinc oxide (ZnO) has a further separate peak at 270 nm. Other investigations have shown that absorbance peaks at 230 and 270 nm in copper-doped ZnO nanoparticles suggest the presence of ZnO^40,48–50^. The production of Ag nanoparticles is shown by the absorbance band at 370 nm^15^. The aqueous extract of *Berberis vulgaris* leaf and root was used to generate nanoparticles of silver, which showed a broad peak in 380–400 nm area^53^. The results of the current study are fully consistent with all the outcomes.

Different parameters, including pH, temperature, reaction duration, and reactant concentration, can be used to optimize the green synthesis of nanoparticle morphological characterization^33,51–53^. Most of these environmental elements that influence nanoparticle synthesis should be identified. Consequently, these aspects can be efficiently addressed to maximize the yield of industrial fabrication of metallic nanoparticles^53^. The reaction's pH has significant effects on the nanoparticles' structure^61^. To be more precise, temperature and pH have an impact on how nucleation centers develop. In order to maximize the synthesis of metal nanoparticles, it is crucial to adjust the pH level since this results in the automatic growth of nucleation centers^62^. Moreover, the size and structural composition of the nanoparticles have been found to be significantly impacted by the pH of the solution^53^. Therefore, the green synthetic nanocomposite was generated at different pHs to determine which plant extract produced the heaviest dry weight of nanocomposite. To generate a green synesthetic nanocomposite of a trimetallic nanocomposite, these aromatic plant extracts (reductants) are separately adjusted at different pHs (5.5, 7, and 14). Then, the precursors that were used are added gradually and equally. For all studied aromatic plant extracts, the optimum response was seen at a pH between 5.5 and 7, as Fig. 1IV illustrates. Moreover, the heaviest dry weight of the generated nanocomposite was obtained using the *Ziziphus spina-christi* extract at all applicable pHs (Fig. 1V). In brief, the largest dry weight of green synthetic nanocomposite was measured at pH-5.5 (0.29 mg/mL), followed by pH-7 (0.25 mg/mL), and pH-14 (0.05 mg/mL) was the lowest. With the exception of other extracts in all applicable screening analyses, the *Ziziphus spina-christi* extract produced the heaviest dry weight of the formed nanocomposite. Therefore, in all additional investigations, the *Ziziphus spina-christi* extract was selected for the green-generated nanocomposite.

**Figure 2:**
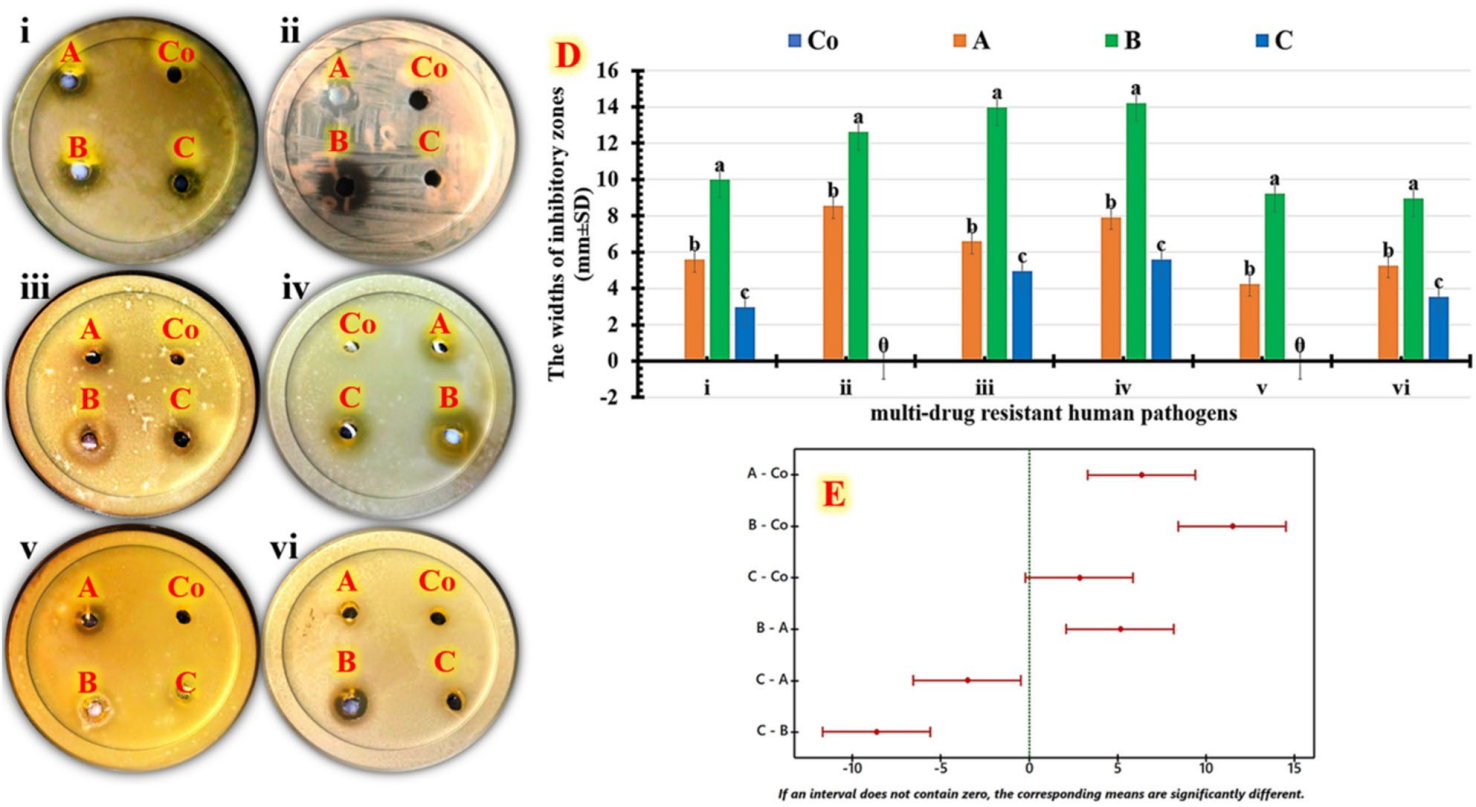
Antimicrobial effects of green synthesized nanocomposite utilizing *Ziziphus spina-christi* extract at all applicable pHs (**A**): pH-5, (**B**) pH-7, and (**C**) pH-14, in comparison to (**Co**): control against (**i**) *Escherichia coli*, (**ii**) *Klebsiella pneumoniae*, (**iii**) *Staphylococcus aureus*, (**iv**) *Bacillus subtilis*, (**v**) *Candida albicans*, and (**vi**) *Candida krusei* using agar-well diffusion analysis. Photos of antimicrobial plates are shown, as well as a chart of the computed inhibition zones (**D**) and simultaneous Tukey tests for mean difference using Tukey–Kramer post-hoc analysis (**E**).

An antimicrobial survey is carried out utilizing the green synthetic nanocomposite, which is prepared using *Ziziphus spina-christi* extract at all applicable pHs. When compared to the free extract (Co), the growth of the evaluated human pathogens was impacted by every nanocomposite created, as demonstrated by the plate photographs (Fig. 2). In brief, the widest inhibitory zone widths (Fig. 2D) were detected at pH 7 against *Bacillus subtilis* (14.21 ± 1.56 mm) and *Staphylococcus aureus* (13.96 ± 2.33 mm). *ANOVA* and *Tukey post-hoc* tests were used to assess the mean values of the computed inhibitory zones to statistically identify the more effective versions. To find significant mean differences, Fig. 2E then displays *Tukey*'s test means for each paired comparison. The adjusted confidence intervals are computed using the Tukey simultaneous tests on a 95% scale. At pH 7 intervals, the green synthesized nanocomposite is devoid of the zero line. This indicates that there are statistically significant differences between the green synthetic nanocomposite at pH 7 and the control group and other tested pHs. The results show statistically significant antimicrobial properties for the tested green synthetic nanocomposite at pH 7.

### Characterization of green synthesized CuO/Ag/ZnO nanocomposite

**Figure 3:**
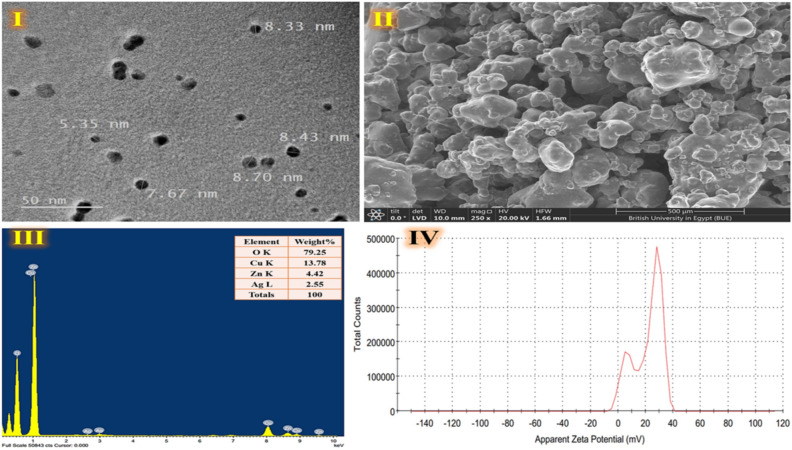
TEM image **(I)**, SEM image **(II)**, TEM–EDX analysis **(III)**, and Zeta potential pattern **(IV)** of green synthesized trimetallic CuO/Ag/ZnO nanocomposite.

TEM imaging of green synthesized nanocomposite is observed with an accelerating voltage of 100 kV. Figure 3I shows the dense, spherical dot-like structure of the green, synthetic trimetallic CuO/Ag/ZnO nanocomposite. This proves the effective development of trimetallic CuO/Ag/ZnO nanocomposite, which is produced in an environmentally friendly manner. The particle size measured on TEM images is used to visualize the real size of nanoparticles, and the result is an average particle size of 7.11 ± 0.67 nm with a narrow particle size dispersion. SEM investigation shows the film surface morphology, which can be characterized as a porosity-free, soft, smooth planar structure (Fig. 3II**)**. Previous studies also used the green chemistry method using extract of *Ocimum basilicum L.,* to generate Ag/doped ZnO-MgO-CaO nanocomposite (59 nm) and spherical and triangular-shaped Ag/doped MgO-NiO-ZnO nanocomposite (30–44 nm); respectively^12,56^. Furthermore, the spherical-shaped of ZnO-Ag nanocomposites (26.02 ± 1 nm) were formed by utilizing a novel, simple, cost-effective, and safe method that involved the utilization of *Stenotaphrum secundatum* extract^13^. Likely, a green technique is employed in a prior study to prepare Ag-doped ZnO nanoparticles (60 nm) utilizing *Tridax procumbens* leaf extract. These nanoparticles show synergistic antimicrobial properties against a variety of human pathogens^8^. As seen in Fig. 3III, EDX mapping verification at multiple sites demonstrates the presence of signals with a highly homogenous distribution on the surface of green synthesized nanocomposite, including O (79.25%), Cu (13.78%), Zn (4.42%), and Ag (2.55%). The study's findings verify that CuO, Ag, and ZnO nanocomposite are effectively synthesized using green techniques. Prior to this, the normal stoichiometric ratio that was employed to generate the trimetallic nanoparticles was not followed, resulting in a compositional atomic ratio of (1:1.46:1.05) of (Cu:Ag:Zn). This could have been brought about by differences in the surface energy of the nanoparticles or by the specific crystallographic orientation of the metal atoms^15^. A further vital characteristic is the ability to measure charge on a surface. The molecular weight of large molecules dissolved in water can be determined using Zeta-potential analyzer. Zeta potential levels rely on a number of factors, including chemical composition and roughness^64^. Zeta potential is a measure of the strength of charge on the surface of particles^64,65^. The stability of an emulsion or nanosuspension can be predicted based on the absolute value of the zeta potential. In order to stabilize the nanocrystal formation (electrostatic repulsion), a high absolute value of zeta potential needs to be achieved. Higher zeta potentials of the nanomaterial suspension predicted the formation of a more stable, non-aggregating particle dispersion. Previous studies found that a suspended particle is deemed stable if its zeta potential is either higher than + 30 mV or lower than − 30 mV^65,66^. According to earlier studies, particles will agglomerate when zeta potential values get closer to 0 mV^67^; nevertheless, for values larger than ± 20 mV, the particles will remain stable and suspended^65^. The green synthetic trimetallic CuO/Ag/ZnO nanocomposite has a zeta-potential of 21.5 ± 5.53 mV, as shown in Fig. 3IV. The large absolute zeta potentials (> 20 mV) of our developed green synthetic nanocomposite suggested long-term stability by reducing vesicle aggregation, indicating that it was stable in a liquid state.

**Figure 4:**
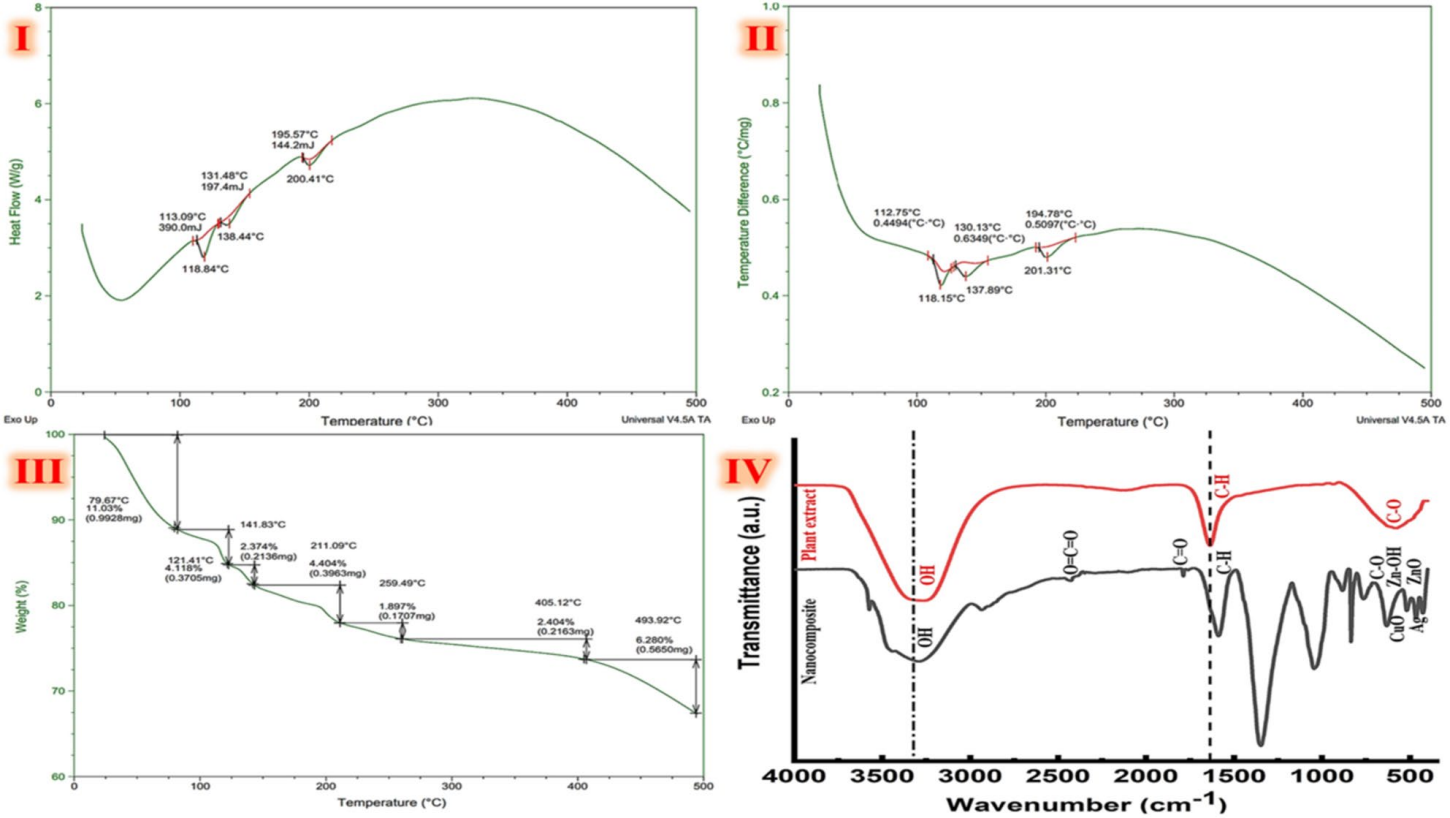
Characterization of green synthesized trimetallic CuO/Ag/ZnO nanocomposite's DSC (**I**), DTA (**II**), and TGA (**III**) curves with FTIR (**IV**) spectrum of green synthesized nanocomposite (black spectrum), and the extract of *Ziziphus spina christi* (red spectrum).

Furthermore, the thermal analysis of green synthesized trimetallic CuO/Ag/ZnO nanocomposite is characterized using TGA, DTA, DSC profiles (Fig. 4). DSC data provides a detailed description of the phase transition of tested nanocomposite. The Tg value is a crucial parameter to describe the stability of the lyophilized nanocomposite. The transition temperature and associated enthalpy drop have an impact on the stability of drug pharmacokinetics. A more tightly constructed nanocomposite is suggested by a greater transient enthalpy. DSC panel indicates that green synthetic trimetallic CuO/Ag/ZnO nanocomposite's transition temperature varied between 100 and 200 °C (Fig. 4I). The characteristic endothermic peaks appear at approximately 118.84 °C, 138.44 °C, and 200.41 °C. These are caused by the release of absorbed water, the breakdown of organic molecule function groups, depolymerization, and decomposition, as well as the dehydration, phase conversion, and full combustion of the organic residue^68,69^. The green synthetic trimetallic CuO/Ag/ZnO nanocomposite's DTA curve (Fig. 4II) displays three exothermic peaks at 112.75, 130.13, and 194.78°C and three endothermic peaks at 118.15, 137.89, and 201.31°C. The heat degradation process is shown in seven phases on the TGA curve in a smooth, stepwise manner (Fig. 4III). While weight losses of 11.03, 4.12, 2.37, 4.404, 1.89, 2.404, and 6.288% accompanied the breakdown of green synthetic trimetallic CuO/Ag/ZnO nanocomposite, are detected at 79.67, 121.41, 141.83, 211.09, 259.49, 405.12, and 493.92°C. The green synthetic trimetallic CuO/Ag/ZnO nanocomposite loses weight in the initial stages due to the evaporation of adsorbed water molecules and humidity. Because of the breakdown of green synthetic trimetallic CuO/Ag/ZnO nanocomposite matrix, the largest weight losses (> 85.92%) occur at temperatures between 0 and 250 °C, because of CuO/Ag/ZnO is crystallinity-related, the final breakdown (10.57%) takes place between 260 and 500°C.

FTIR spectra of *Ziziphus spina christi* extract and CuO/Ag/ZnO nanocomposite specimens are shown in Fig. 4IV. The spectrum of CuO/Ag/ZnO nanocomposite exhibits peaks at around ν 700–400 cm^−1^; in contrast to the extract bonds of *Ziziphus spina christi* spectrum. This can be attributed to the interactions that Ag has with metal oxides like ZnO or CuO. The green synthesized spectrum of CuO/Ag/ZnO nanocomposite shows distinct peaks at ν 630 cm^−1^ and peaks at approximately ν 500–420 cm^−1^, which are related to the stretching vibrations of CuO and Zn–O, respectively^37–39^. Air humidity most likely influenced the sample measurement. A spectra band of ν 3600–3500 cm^−1^ is where O–H bond occurs^70^. Consequently, the signal at ν 3478 cm^−1^ is associated with inter-hydrogen bonding that is present in both the plant extract and nanocomposite spectra represents –OH groups and water molecules. The stretching frequency of the extract's phenolic O–H, which serves as a reducing and capping ligand, is responsible for the broad peak at ν 3354–1606 cm^−1^. The stretching of carbon dioxide O=C=O bonds is also responsible for the peak at ν 2351 cm^−1^. The additional clear peak is especially visible at ν 1520 cm^−1^ which is the vibrational frequency of a C=O bond and may indicate the presence of organic residues. Furthermore, there is a peak at ν 1427 cm^−1^, which could be related to the O–H bonds in carboxylic acid bending. The stretching vibration of C=O polyphenols may be explained by strong peak at ν 1392 cm^−1^. Aromatic C–O and N–H stretching vibrations from phenolic groups were responsible for the strong peaks at 1268 and ν 1076 cm^−1^; respectively. The stretching of C–O bonds in primary alcohols is connected to the peak detected at ν 1113 cm^−1^. Overall, FT-IR spectrum of CuO/Ag/ZnO exhibits that; it is coated with active phytoconstituents, mainly O–H, C=O, and C–N residues of alkaloids and phenolic derivatives. To stabilize the resulting CuO/Ag/ZnO nanocomposite, O–H, C=O, and C–N residues might form bonds with metals by covering their surfaces and decreasing agglomeration^69,71^. Many studies have reported that a variety of biomolecules found in the *Ziziphus spina christi* extract are responsible for the stabilization and reduction of the green synthesized nanocomposite. The existence of several functional groups linked to active phytochemicals such as phenolic acids, flavonoids, aromatic compounds, etc*.* is shown by FTIR analysis of trimetallic Zn/Cu/Ag NCs that are synthesized from the leaf extract^72^. These groups have been suggested to be responsible for the generation of the trimetallic nanocomposite as well as the reduction of metal precursors and subsequent stabilization^61^. The phytochemicals in the extract, primarily flavonoids and phenolic acids, may decrease metal ions by donating electrons, resulting in the generation of metal nanoparticles. Furthermore, this may prevent the particles from aggregating by binding to the surface of the nanoparticle, forming a barrier that reduces surface energy and stabilizes the particles. Further oxidation of the nanoparticles could be inhibited by the carboxyl and hydroxyl groups binding to the metal ions on their surface, protecting the structural integrity of the particles^11,51^. Our FTIR results clearly show the presence of flavonoids and phenolic acids, which are responsible for the development of the green synthetic trimetallic CuO/Ag/ZnO nanocomposite.

### Statistical optimization of the yield of green synthesized nanocomposite

In general, green synthesized nanocomposites show promise as antibacterial and anticancer agents for safer, more effective, and inexpensive medications or drug delivery systems. The various sizes, forms, dispersions, and stability of the generated nanocomposites are associated with the presented metabolites^40^. Green synthesized procedures for nanocomposite, in particular, have a number of delicate factors^63,64^. Several factors that influence the yield shape and size control include the concentrations of plant extract and precursors, as well as the ratio of precursors to other reaction parameters, including temperature, pH, agitation, and incubation time^54,65^. Worldwide, scientific investigations are being carried out to find out more about how temperature affects nanoparticles^33,54^. The main element that alters the size, shape, and degree of synthesis of the nanoparticles is the temperature^36^. Temperature-dependent modifications can be made to the synthesized nanoparticles' rod, spherical, octahedral platelet, triangular, and spherical shaped structure. Additionally, when the temperature improves, the reaction response rate increases the nucleation center development^54,65^. Conversely, the most important variable influencing the yield, size, and shape of nanoparticles generated during the synthesis of green nanoparticles is the reaction time^3,62^. According to EL-Moslamy et al., reported that reaction time is critical to produce various nanoparticles and nanocomposites. Therefore, three primary parameters that influence a nanoparticle's shape and structure are temperature, pH, and reaction time^53,54^. Until now the utilization of *Ziziphus spina christi* extract to optimize the conditions of green synthesized trimetallic (CuO/Ag/ZnO) nanocomposite statistically according to regulated conditions remains unexplored. In this study, a two-step experimental strategy known as *Plackett–Burman* and *Taguchi* designs is utilized to analyze the parameters influencing the green synthesized reaction to maximize the nanocomposite's green synthesized yield.

#### Plackett–Burman design

The best parameters for maximizing the dry weight of nanocomposite solutions are identified by using this qualitative and quantitative screening method employing green-synthesized reaction variables. The chosen experiments are utilized to identify the essential elements for the green synesthetic nanocomposites, determine the appropriate ratio, and create a mathematical model, that could be applied to the prediction procedure. The 12 experiments involved screening several components of green-synthetic reaction and exploring each one at two different levels: high (+ 1) and low (− 1), together with a dummy factor used to assess the experiment's standard error. The experiments are completed, and green synthesized nanocomposite’s dry weights are recorded (Table 4). Excel 2016 and *Minitab 18* are the tools utilized for statistical analysis and graph plotting. As indicated by Table 4 the nanocomposite's highest dry weight was 0.78 mg/mL (run 12) and 0.65 mg/mL (run 8); in contrast, the lowest dry weight is 0 mg/mL that recorded at runs 5 and 7.

**Table 4:** Seven distinct factors (F1: plant extract concentrations, F2: precursor concentrations, F3: precursor ratio, F4: reaction agitation, F5: reaction temperature, F6: reaction pH, and F7: incubation period) and their impact on the dry weight production efficiency of green synthesized nanocomposites employing the *Plackett–Burman* design.

Std order	Run order	Pt type	Blocks	F1	F2	F3	F4	F5	F6	F7	Dry weight (mg/mL)	Predicted results (mg/mL)
6	1	1	1	1	1	1	− 1	1	1	− 1	0.31	0.33
2	2	1	1	1	1	− 1	1	− 1	− 1	− 1	0.09	0.10
7	3	1	1	− 1	1	1	1	− 1	1	1	0.07	0.12
12	4	1	1	− 1	− 1	− 1	− 1	− 1	− 1	− 1	0.41	0.44
8	5	1	1	− 1	− 1	1	1	1	− 1	1	0	− 0.02
1	6	1	1	1	− 1	1	− 1	− 1	− 1	1	0.47	0.48
3	7	1	1	− 1	1	1	− 1	1	− 1	− 1	0	− 0.02
11	8	1	1	− 1	1	− 1	− 1	− 1	1	1	0.65	0.60
4	9	1	1	1	− 1	1	1	− 1	1	− 1	0.35	0.31
5	10	1	1	1	1	− 1	1	1	− 1	1	0.13	0.12
9	11	1	1	− 1	− 1	− 1	1	1	1	− 1	0.27	0.28
10	12	1	1	1	− 1	− 1	− 1	1	1	1	0.78	0.80

The effect of each independent variable on the response is ascertained by analysis of variance (*ANOVA*), where P < 0.05 was deemed statistically significant. Table 4 shows the results of equation's fitness evaluation using the multiple correlation coefficient (R^2^) and adjusted R^2^. In the overall design, the p value indicates the significance of each independent variable. Larger t-values and smaller p-values (prob > F < 0.05) are associated with greater coefficient influence on the response. The model's overall performance is also estimated using the coefficient of determination (R^2^) and the adjusted-R^2^ (adj-R^2^) value, which ideally should agree with R^2^ value (less than 2%). A stronger model with better response prediction is indicated by R^2^ value closer to 1^75,76^. The presented data shows model R^2^ and adj-R^2^ values for the bio-fabrication reaction of the green synthesized nanocomposite, which are 98.58%, and 96.10%; respectively (Table 5). According to these findings, the model can account for 98.58% of response data variability, with a 1.42% chance that noise is to blame for the variation. Additionally, a high adj-R^2^ value showed that the model was precise and that there is a strong correlation between the experimental and anticipated findings. *ANOVA* summary typical of experimental Plackett–Burman tests indicated that the model was highly significant, due to the low probability value (p value ~ 0.05). Regarding the green synesthetic nanocomposite's dry weight (mg/mL), each of these components showed an acceptable adjustment (Table 5).

**Table 5:** *Plackett-Berman* statistical analysis used to optimize factors to increase the production efficiency of green nanocomposites.

Source	DF	Adj SS	Adj MS	F-value	P-value
Model	7	0.692925	0.098989	39.73	0.002
Linear	7	0.692925	0.098989	39.73	0.002
Concentrations of plant extract	1	0.044408	0.044408	17.82	0.013
Concentrations of precursor	1	0.088408	0.088408	35.48	0.004
Ratio of precursors	1	0.106408	0.106408	42.71	0.003
Reaction agitation	1	0.243675	0.243675	97.80	0.001
Reaction temperature	1	0.025208	0.025208	10.12	0.034
Reaction pH	1	0.147408	0.147408	59.16	0.002
Incubation time	1	0.037408	0.037408	15.01	0.018
Error	4	0.009967	0.002492	–	
Total	11	0.702892	–	–	–

R-sq = 98.58%, R-sq(adj) = 96.10%, and R-sq(pred) = 87.24%.

**Figure 5:**
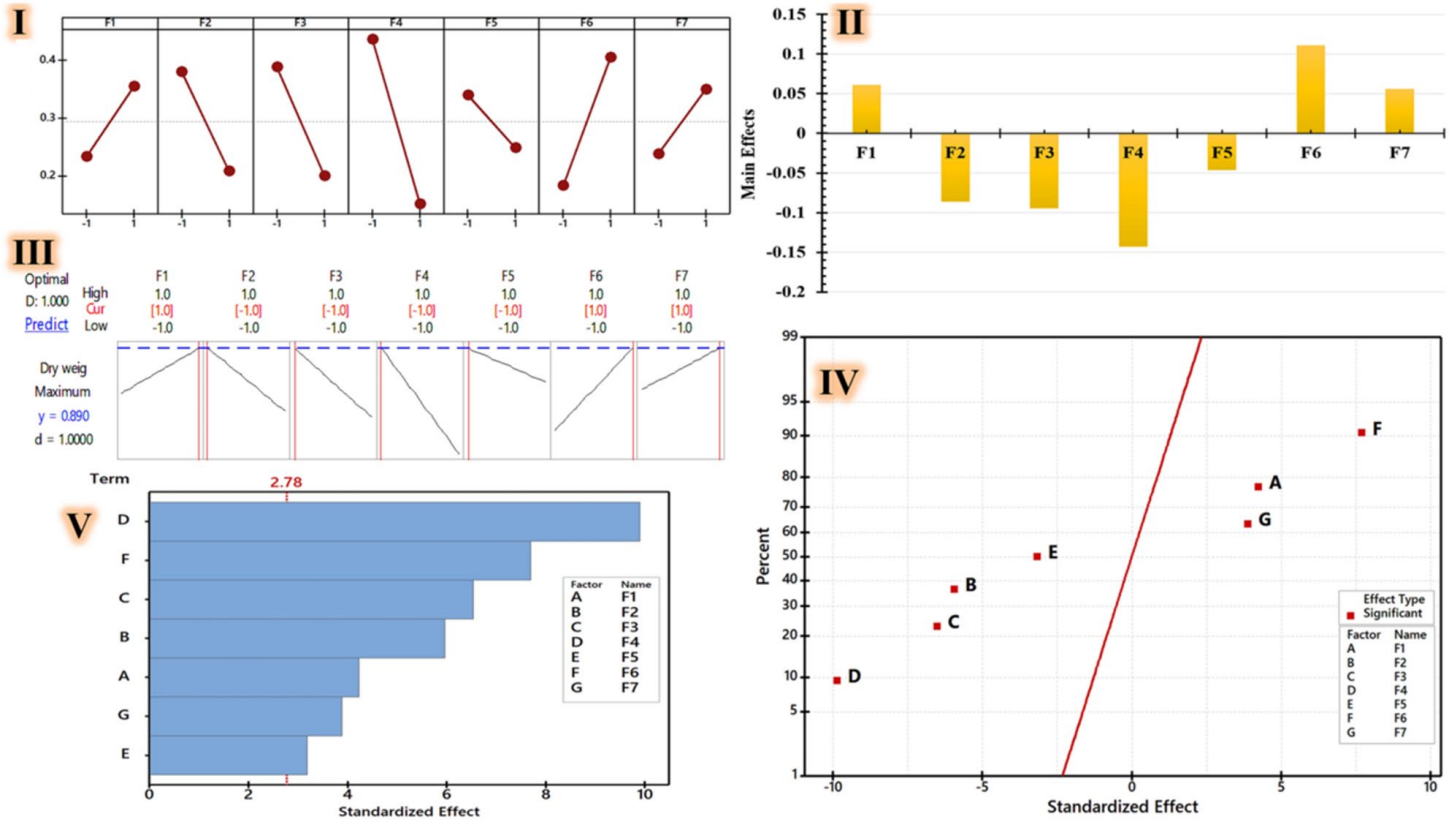
Model summary of the factorial regression for the green synthetic nanocomposite (g/ml) for the investigated variables: plant extract concentrations (**F1**), precursor concentrations (**F2**), precursor ratio (**F3**), reaction agitation (**F4**), reaction temperature (**F5**), reaction pH (**F6**), and incubation period (**F7**) via the following parameters: the main effect plot (**I** and **II**), the standardized effect using normal plot (**IV**), Pareto chart of the standardized effects (**V**), and a response optimizer with a maximum outcome and optimal values for these variables (**III**) of each variable.

As shown in Fig. 5I, II, and IV nanocomposite's yield is affected by the minimized values of precursor concentrations (F2), precursor ratio (F3), reaction agitation (F4), and reaction temperature (F5) factors, alongside the maximized values of plant extract concentrations (F1), reaction pH (F6), and incubation period (F7). The production efficiency of green synthesized nanocomposites is statistically significantly impacted by all evaluated parameters. As illustrated in Fig. 5V concentrations of plant extract (F1), concentrations of precursors (F2), ratio of precursors (F3), reaction agitation (F4), reaction pH (F6), and incubation time (F7); are the main factors that influence the production efficiency of green synthesized nanocomposites, more so than reaction temperature (F5). Figure 5I illustrates the principal impacts of every variable under investigation on the nanocomposite's dry weight. These main effects describe the average differences for each variable between its low and high values. Except for F2, F3, F4, and F5 factors, which vary dramatically between high and low levels, suggesting their impact on amplifying the response at low levels. As a factor rises from a low to a high level, the response always increases when the major effect of the factor is positive (F1, F6, and F7). Because it predicts the maximum dry weight of the nanocomposite using optimal parameters (Fig. 5III) to determine individual effectiveness, the optimizer tool in *MINITAB 18.0* was utilized to solve Eq. (7). Equation (7) indicates that a first-order polynomial model that serves as the starting point for the mathematical modeling of the PBD is used to verify the reaction by calculating the average dry weight of green synthesized nanocomposite. The green synthesized nanocomposites are verified by means of the ideal conditions expected for the green reaction, and the results are compared with those recorded under the baseline settings. By using this optimization process, the nanocomposite's dry weight increases from 0.29 to 0.89 mg/mL, i.e. a 3.06-fold increase.

7 $$ Dry \, weightof \, green \, synthesized \, nanocomposite\left( {\text{mg/mL}} \right) \, = \, 0.{2942 } + \, 0.0{6}0{\text{8 F1 }} - \, 0.0{\text{858 F2 }} - \, 0.0{\text{942 F3 }} - \, 0.{\text{1425 F4 }} - \, 0.0{\text{458 F5 }} + \, 0.{11}0{\text{8 F6 }} + \, 0.0{\text{558 F7}}. $$

#### Taguchi statistical method

A cost-efficient and attractive tool for optimizing and generating excellent industrial production processes has been developed by *Genichi Taguchi* model. According to the requirements of the experiment, several arrays included in *Taguchi's* model can be employed. Orthogonal arrays (OAs) have designs indicated by Ln (mP), where n signifies the total number of sections, m denotes the number of parameter levels, and P is the total number of parameters^69,70^. This work is the first to employ the *Taguchi* experimental design to statistically optimize the conditions of green synthesized trimetallic CuO/Ag/ZnO nanocomposite qualities using *Ziziphus spina christi* extract. *Taguchi's* L27 (3^7) orthogonal array design is utilized to optimize the yield of a green trimetallic CuO/Ag/ZnO nanocomposite. The yield and S/N ratio values of green trimetallic CuO/Ag/ZnO nanocomposite are determined by conducting 27 trials with seven parameters classified according to L27 (3^7) OA design, as indicated in Table 6. The ideal combination of the responses of green trimetallic CuO/Ag/ZnO nanocomposite is designed by experimentation using *Taguchi* model. To identify the best combination of the evaluated factors, the data is examined using statistical techniques, including regression analysis and *ANOVA*. The biggest and smallest yields of green trimetallic CuO/Ag/ZnO nanocomposite values (0.04 and 1.42 mg/mL) are demonstrated in experimental No. 25 and No. 12; respectively. Table 6 displays the structure of *Taguchi's* orthogonal robust structure, as well as the measurement outcomes. The quality feature that deviates from the intended value is measured using S/N ratio data obtained from *Taguchi* method. S/N ratios vary based on the green trimetallic CuO/Ag/ZnO nanocomposite yield values (Table 6). The S/N ratio and green trimetallic CuO/Ag/ZnO nanocomposite yield values determined by *Taguchi's* equation (Eq. 3) are displayed in Table 6.

**Table 6:** Experimental setup that uses *Taguchi's* L27 (3^7) orthogonal array design to maximize the manufacturing efficiency of green trimetallic CuO/Ag/ZnO nanocomposite.

Experimental runs	F1	F2	F3	F4	F5	F6	F7	Dry weight (mg/ml)	S/N ratio (dB)
L1	1	1	1	1	1	1	1	0.49	− 6.19608
L2	1	1	1	1	2	2	2	0.48	− 6.37518
L3	1	1	1	1	3	3	3	0.64	− 3.8764
L4	1	2	2	2	1	1	1	0.59	− 4.58296
L5	1	2	2	2	2	2	2	0.67	− 3.4785
L6	1	2	2	2	3	3	3	0.74	− 2.61537
L7	1	3	3	3	1	1	1	0.91	− 0.81917
L8	1	3	3	3	2	2	2	0.89	− 1.0122
L9	1	3	3	3	3	3	3	0.96	− 0.35458
L10	2	1	2	3	1	2	3	0.89	− 1.0122
L11	2	1	2	3	2	3	1	0.76	− 2.38373
L12	2	1	2	3	3	1	2	1.42	3.045767
L13	2	2	3	1	1	2	3	0.29	− 10.752
L14	2	2	3	1	2	3	1	0.16	− 15.9176
L15	2	2	3	1	3	1	2	0.81	− 1.8303
L16	2	3	1	2	1	2	3	0.54	− 5.35212
L17	2	3	1	2	2	3	1	0.42	− 7.53501
L18	2	3	1	2	3	1	2	1.26	2.007411
L19	3	1	3	2	1	3	2	0.42	− 7.53501
L20	3	1	3	2	2	1	3	1.04	0.340667
L21	3	1	3	2	3	2	1	1.02	0.172003
L22	3	2	1	3	1	3	2	0.85	− 1.41162
L23	3	2	1	3	2	1	3	1.29	2.211794
L24	3	2	1	3	3	2	1	1.27	2.076074
L25	3	3	2	1	1	3	2	0.04	− 27.9588
L26	3	3	2	1	2	1	3	0.69	− 3.22302
L27	3	3	2	1	3	2	1	0.56	− 5.03624

The investigated variables coded as; plant extract concentrations (F1), precursor concentrations (F2), precursor ratio (F3), reaction agitation (F4), reaction temperature (F5), reaction pH (F6), and incubation period (F7).

The mean S/N ratio for each parameter level is reported, and Table 6 displays the S/N response table for yield of the green trimetallic CuO/Ag/ZnO nanocomposite. Both an *ANOVA* and an F-test can be used to assess the experimental data (Table 7). Our chosen model suits the experimental data well, as evidenced by its R^2^ of 97.36%. So, both the model and its parameters were highly significant (P < 0.0001). The model's F-value stands at 100.33, and the significance F-value is 1.19 E−13 (Table 7).

**Table 7:** Results of *Taguchi* design experimental design analysis, which is utilized to optimize the production efficiency of green synesthetic nanocomposite.

	Coefficients	Standard error	t Stat	P-value	
Intercept	0.154444	0.082728	1.86689	0.077426	
Concentrations of plant extract	0.045	0.015451	2.912376	0.008932	
Concentrations of precursor	− 0.04944	0.015451	− 3.20002	0.004714	
Ratio of precursors	− 0.04111	0.015451	− 2.66069	0.015442	
Reaction agitation	0.282222	0.015451	18.26527	1.65E−13	
Reaction temperature	0.203333	0.015451	13.15963	5.37E−11	
Reaction pH	− 0.195	0.015451	− 12.6203	1.1E−10	
Incubation time	0.05	0.015451	3.235974	0.004349	

	df	SS	MS	F	Significance F
ANOVA					
Regression	7	3.018217	0.431174	100.3344	1.18595E−13
Residual	19	0.08165	0.004297	–	–
Total	26	3.099867	–	–	–

Multiple R = 0.98, R^2^ = 0.97, adjusted R^2^ = 0.96, and standard error = 0.065.

**Figure 6:**
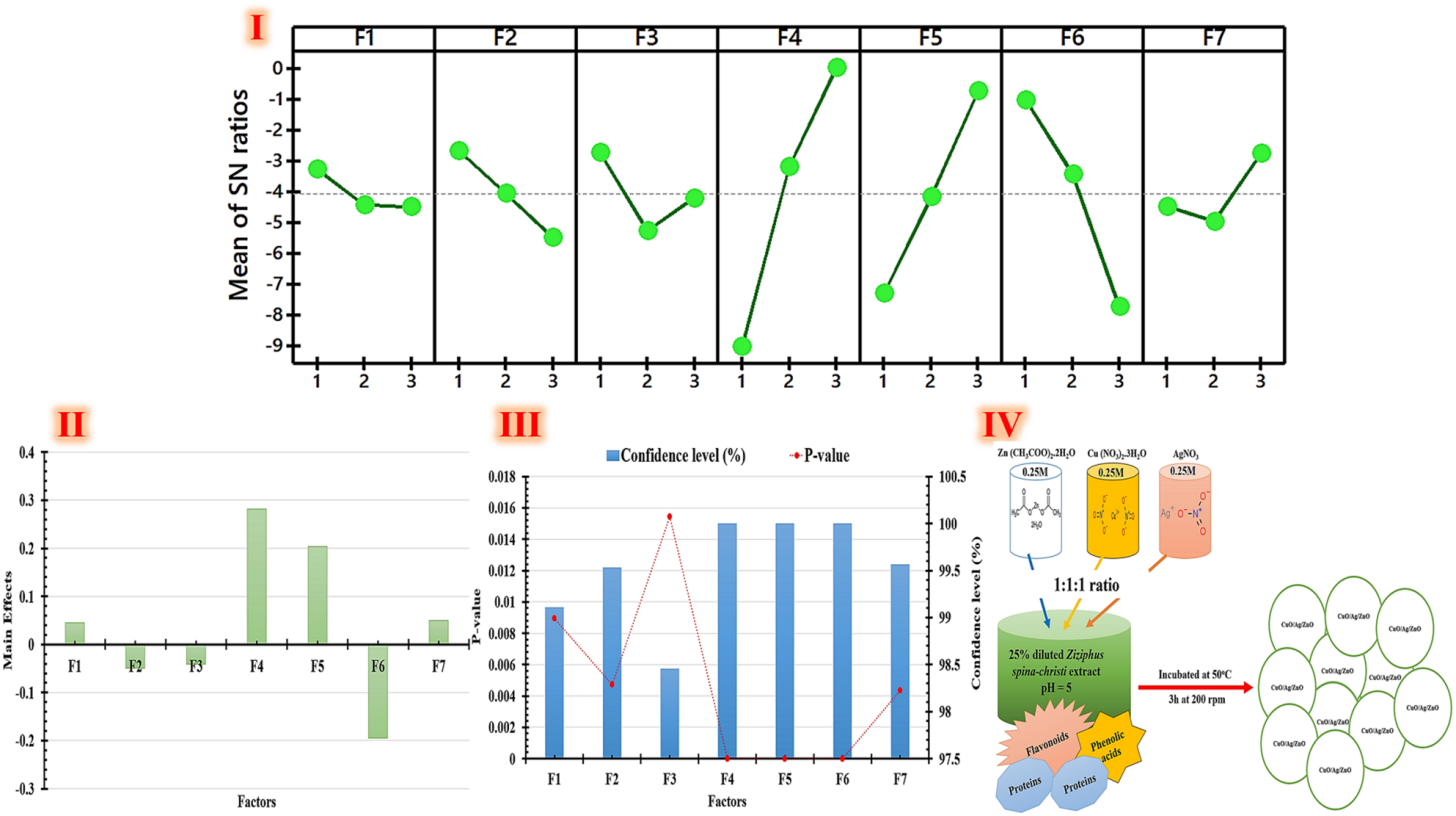
Characteristics of Taguchi's experimental results: (**I**) the larger-the-better main effects plot for S/N ratios; (**II**) the main effects plot for means of the production efficacy of green synesthetic nanocomposite; (**III**) the p-values and confidence level (%) of each factor in the yield of green synesthetic nanocomposite, and **(IV)** Schematic diagram of the green synthetic trimetallic CuO/Ag/ZnO nanocomposite employing 25% diluted *Ziziphus spina-christi* extract (pH = 5) as reducing/capping agent and 0.25 M AgNO, Cu(NO_3_)_2_.3H_2_O, and Zn(CH_3_COO)_2_.2H_2_O as precursors. The green synthesized trimetallic CuO/Ag/ZnO nanocomposite coated with active phytoconstituents, mainly O–H, C=O, and C–N residues of alkaloids and phenolic derivatives.

It is shown that, the suggested model is adequate by the residuals found above and below zero line of the residual plot. A straight-line distribution is seen in the residual plots, which suggests the model fits the results effectively (Fig. 6). The end confidence level (%) and P-values of each factor indicated that F4, F5, and F6 are significant factors, followed by F7, F2, F1, and F3 (Fig. 6III). This orthogonal array model is represented by equation No. 8, which also explains the yields of green trimetallic CuO/Ag/ZnO nanocomposite and the relationships between each of the seven elements. As illustrated in Fig. 6I, the final rankings have the largest S/N ratio value (bigger is better) based on the *ANOVA* analysis of S/N ratio value and the factor level calculation of the main impact for this dry weight (Table 7). For the key effects obtained all through the optimization trial runs, a primary impact graphic was drawn (Fig. 6II).

8 $$ Dry \, weightof \, the \, green \, synesthetic \, nanocomposite\left( {\text{mg/mL}} \right) \, = \, 0.{15} + \, 0.0{45}{\mathbf{F1}} - 0.0{49}{\mathbf{F2}} - 0.0{41}{\mathbf{F3}} + 0.{28}{\mathbf{F4}} + 0.{2}0{3}{\mathbf{F5}} - 0.{195}{\mathbf{F6}} + \, 0.0{5}{\mathbf{F7}}. $$

To get the highest yield of the green synthesized trimetallic CuO/Ag/ZnO NCs, this approach recommends the optimal combination of the investigated parameters. For green trimetallic CuO/Ag/ZnO nanocomposite, S/N ratio indicates that the following are the ideal conditions: F1 at level 1, F2 at level 1, F3 at level 1, F4 at level 3, F5 at level 3, F6 at level 1, and F7 at level 3. The last stage is to predict and confirm the improvement of quality profile using the ideal level of design parameters after the optimal level has been determined. According to Eq. (4), it is possible to compute the predicted S/N ratio using design parameters at their optimal level. The pH of 25% diluted *Ziziphus spina-christi* extract is adjusted to 5 to achieve the highest dry weight possible for green trimetallic CuO/Ag/ZnO nanocomposite. This extract is then titrated slowly using (0.25M AgNO_3_, Cu(NO_3_)_2_.3H_2_O, and Zn(CH_3_COO)_2_.2H_2_O), which are prepared at 1:1:1 ratio. This reaction is incubated at 50 °C and agitated for 3h at 200 rpm, after this titration phase (Fig. 6IV). The green trimetallic CuO/Ag/ZnO nanocomposite is optimized statistically under controlled conditions using *Ziziphus spina christi* extract, resulting in an estimated yield of 1.65 mg/ml and a predicted S/N ratio of roughly 7.79 dB. Lastly, the comparison of data using *Placket Burman* strategy and *Taguchi* approach shown that it is feasible to efficiently raise and enhance the yield of green synthetic trimetallic CuO/Ag/ZnO nanocomposite. Compared to *Plackett Burman* strategy and basal condition, the maximum green synthetic trimetallic CuO/Ag/ZnO nanocomposite yield (1.65 mg/mL) may be increased by 1.85 and 5.7 times; respectively by applying *Taguchi* strategy.

### Antimicrobial potency of green trimetallic CuO/Ag/ZnO nanocomposite

**Figure 7:**
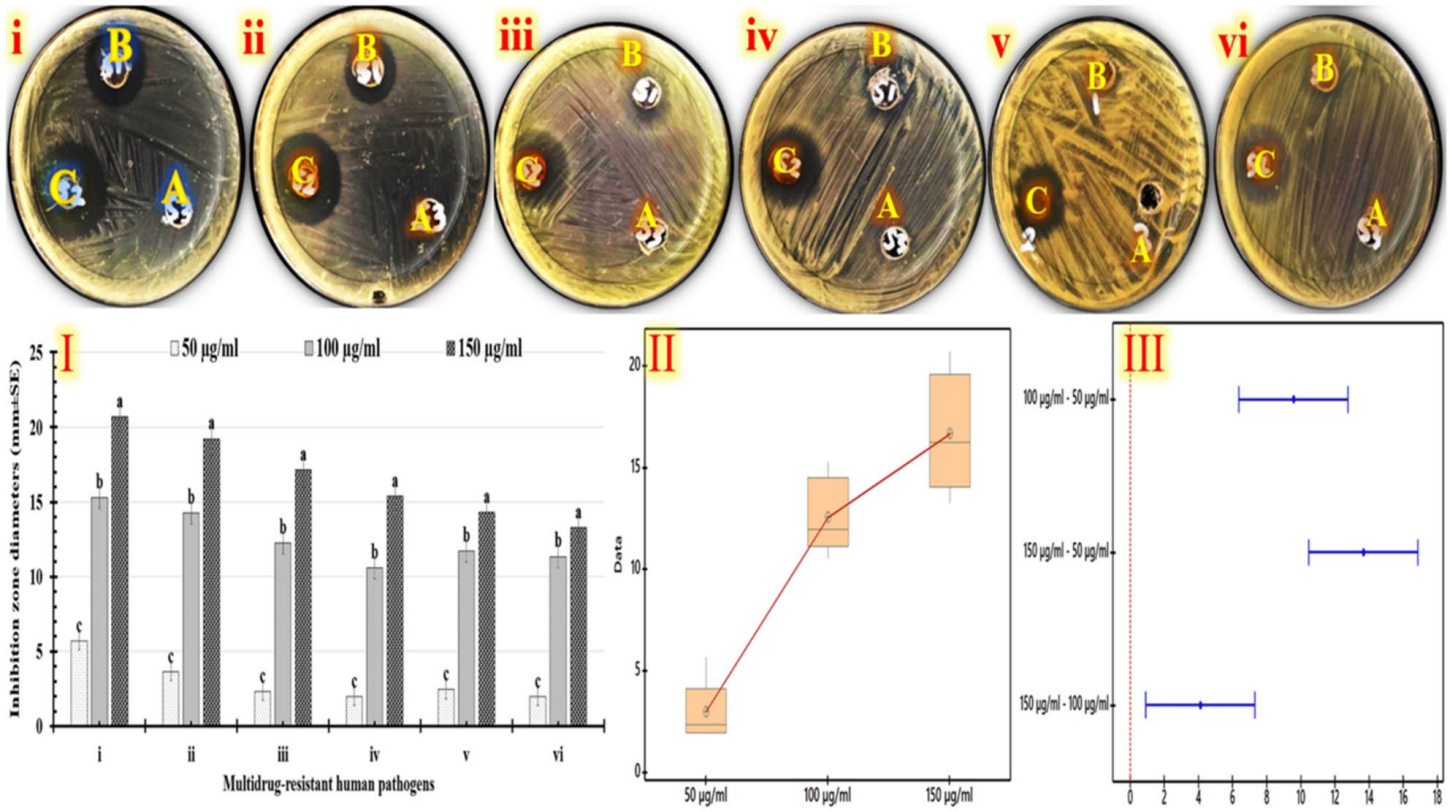
Antimicrobial efficacy results for tested doses of green trimetallic CuO/Ag/ZnO nanocomposite labeled (**A**: 50 µg/mL, **B**: 100 µg/mL,** C**: 150 µg/mL) against various multidrug-resistant human pathogens (**i**: *Escherichia coli,*** ii**: *Klebsiella pneumoniae,* **iii**: *Staphylococcus aureus,*** iv**: *Bacillus subtilis,*** v**: *Candida albicans,* and **vi**: *Candida krusei*). Photographs depict an Agar-well diffusion investigation. Chart displays the computed inhibition zones (**I**), box-plot graph (**II**) displays the inhibitory value distributions corresponding to the tested doses; and simultaneous results for analyzing the overall group's difference (**III**) via *Tukey–Kramer post-hoc* analysis. Means that don't have the same letter differ greatly.

Human infections with antibiotic-resistant microbes are a major cause of death worldwide^79^. Accordingly, a number of antimicrobial nanostructures have been generated recently^80^. So, our study investigated the antimicrobial qualities of different doses of optimized yield of green synthesized trimetallic CuO/Ag/ZnO nanocomposite. Initially, the antimicrobial activity of evaluated doses (50, 100, and 150 µg/mL) is evaluated using agar-well-diffusion method (Fig. 7). The inhibitory zone widths of tested doses of green trimetallic CuO/Ag/ZnO nanocomposite against multidrug-resistant human pathogens are determined. Generally, the largest inhibitory zone widths are recorded by using different doses of the green trimetallic CuO/Ag/ZnO nanocomposite against *Gram*-negative bacteria (Fig. 7i,ii), and *Gram-*positive bacteria (Figs. 7iii, and 8iv), followed by yeast cells (Fig. 7v,vi). There are differences in the affected doses for each human pathogen that has been studied, as seen in Fig. 7I. The results of Table 8 demonstrate that *Escherichia coli* that are treated with 150 µg/mL of green trimetallic CuO/Ag/ZnO nanocomposite shows the largest inhibitory zone widths (20.68 ± 3.54 mm), followed by *Klebsiella pneumoniae* (19.22 ± 1.41 mm), and *Staphylococcus aureus* (17.14 ± 1.98 mm). Additionally, *Bacillus subtilis* (15.39 ± 3.52 mm), *Candida albicans* (14.32 ± 2.54 mm), and *Candida krusei* (13.29 ± 4.22 mm) show the narrowest inhibitory zones, when exposed to 150 µg/mL of green trimetallic CuO/Ag/ZnO NC. Ag-ZnO nanocomposites (75 nm) generated from *fenugreek* leaf extract at a dosage of 20 mg/mL are found to have antimicrobial properties against several human diseases in a previous study. In the agar diffusion method, the inhibition zone diameter for *Escherichia coli* is 12.5 ± 0.707 mm, for *Staphylococcus aureus* is 13.5 ± 0.707 mm, and for *Candida albicans* is 10.5 ± 0.707 mm^81^. But herein, *Escherichia coli* treated with 150 µg/mL of green trimetallic CuO/Ag/ZnO nanocomposite demonstrate the highest inhibitory zone widths (20.68 ± 3.54 mm), followed by *Staphylococcus aureus* (17.14 ± 1.98 mm) and *Candida albicans* (14.32 ± 2.54 mm). Our results show that Ag-ZnO nanocomposites have less impact on *S. aureus* and *E. coli*. Other reports on the antimicrobial abilities of Ag, ZnO, and CuO nanoparticles generated from various plant extracts have also been reported previously^11,74–76^. Numerous nanoparticles with antibacterial qualities have also been demonstrated in other studies, which include silica, iron oxide, copper oxide, magnesium oxide, titanium dioxide, silver, zinc oxide, and cerium dioxide. The capacity of nanomaterials to limit microbial development depends on the layers of the pathogen's cell wall or membrane structure. The synthesized nanostructure's size, shape, and core–shell morphology, which provide a high surface-area-to-volume ratio, also have an impact on the proliferation of microbes^17,82,85^.

**Figure 8:**
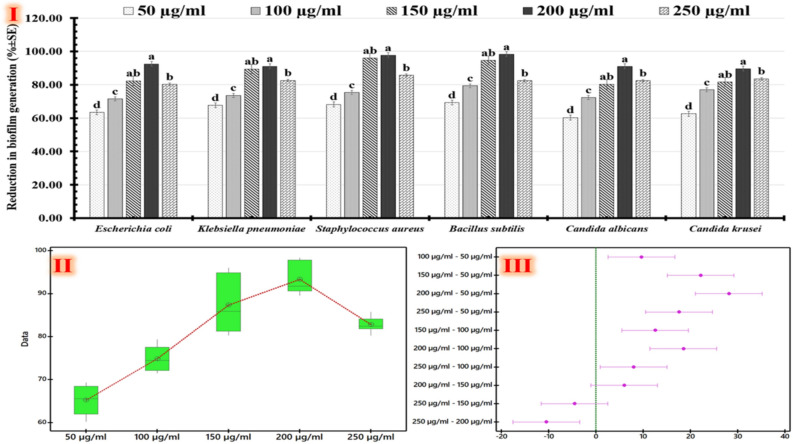
Reduction in biofilm generation of the tested human pathogens using a biofilm inhibition assay. Chart shows the percentage of biofilm reduction (**I**), box-plot graph shows biofilm reduction value distributions corresponding to drug dosages via Tukey–Kramer post-hoc analysis (**II**), and simultaneous Tukey results appearing the overall group's difference (**III**).

**Table 8:** Antimicrobial efficacy results for the tested doses of green trimetallic CuO/Ag/ZnO nanocomposite labeled A: 50 µg/mL, B: 100 µg/mL, and C: 150 µg/mL) measured against various multidrug-resistant human pathogens using an agar-well diffusion.

Multidrug-resistant human pathogens	Inhibition zone diameters (mm ± SD)		
	50 µg/ml	100 µg/ml	150 µg/ml
*Escherichia coli*	5.69 ± 1.25c	15.29 ± 2.87b	20.68 ± 3.54a
*Klebsiella pneumoniae*	3.65 ± 0.33c	14.26 ± 2.58b	19.22 ± 1.41a
*Staphylococcus aureus*	2.33 ± 0.98c	12.25 ± 4.25b	17.14 ± 1.98a
*Bacillus subtilis*	1.98 ± 0.99c	10.58 ± 2.47b	15.39 ± 3.52a
*Candida albicans*	2.45 ± 1.07c	11.72 ± 0.98b	14.32 ± 2.54a
*Candida krusei*	1.98 ± 0.14c	11.35 ± 2.57b	13.29 ± 4.22a

R-sq = 89.60%, R-sq(adj) = 88.22%, and R-sq(pred) = 85.03%. The data is represented as the mean of inhibition zone diameters (millimeters) ± standard deviation (mm ± SD). Statistical significance is reached at p ≤ 0.05 for the differences. Means that don't have the same letter differ greatly.

The *ANOVA*, and *Tukey–Kramer post-hoc* analysis is employed to demonstrate the inhibitory value distributions that correspond to tested doses. Furthermore, data about the correlation between tested products and antimicrobial effectiveness is grouped using statistical clustering. So, the inhibitory effect distributions that match tested treatments are displayed on comparable interval and box plot graphs (Fig. 7II, III). The *Tukey–Kramer post-hoc* results show that, out of all the treatments that are assessed, 150 µg/mL of green trimetallic CuO/Ag/ZnO nanocomposite have the highest anti-biofilm value. A boxplot that displays the significant mean differences is produced for each paired comparison using the means of *Tukey's* test. As can be seen in the box-plot graph (Fig. 7II), there are significant antimicrobial variations among all tested doses of green trimetallic CuO/Ag/ZnO nanocomposite. Especially, 150 µg/mL of green trimetallic CuO/Ag/ZnO nanocomposite have the highest inhibitory values based on *Tukey–Kramer post-hoc* results. On 95% scale, the modified confidence intervals are computed using *Tukey* simultaneous tests. Due to the absence of zero line in the intervals for formulation with the highest efficacy, 150 µg/mL of green trimetallic CuO/Ag/ZnO nanocomposite, whose mean values are shown in Fig. 7III, shows significant differences. All these results indicate that 150 µg/mL of green trimetallic CuO/Ag/ZnO nanocomposite have the strongest antimicrobial properties, compared to all doses that are tested.

Spectrophotometric antibiofilm assay is used to assess the antimicrobial efficacy of green trimetallic CuO/Ag/ZnO nanocomposite with several doses ranging from 50 to 250 µg/mL, against all tested human pathogens. The percentage of biofilm reduction is utilized to determine doses' in vitro efficacy to prevent pathogen growth (Fig. 8). The antimicrobial chart depicts 200 µg/mL dose's strong antagonistic antimicrobial effects against all pathogens tested (Fig. 8I). Additionally, tested *Gram*-positive have the highest antimicrobial effect more than tested *Gram*-negative, and yeast cells. The highest percentage of antibiofilm after treatment with 200 µg/mL of green trimetallic CuO/Ag/ZnO nanocomposite are of 98.31 ± 0.98, and 97.68 ± 1.11% that are recorded against tested *Gram*-positive pathogens *e.g. Bacillus subtilis*, and *Staphylococcus aureus;* respectively (Table 9). Additionally, the modest percentage of antibiofilm are recorded against *Escherichia coli* (92.45 ± 1.41%), *Klebsiella pneumoniae* (91.07 ± 1.09%), *Candida albicans* (90.99 ± 0.87%), *Candida krusei* and (89.59 ± 0.15%), as seen in Table 9. To statistically ascertain whether doses are more effective, the mean values of computed antibiofilm percentages are assessed using *ANOVA* and *Tukey post-hoc test*, Fig. 8II, III. Furthermore, the correlation data between tested doses and antimicrobial effectiveness is grouped using statistical clustering. So, the inhibitory effect distributions that match the tested treatments are displayed on comparable interval and box plot graphs. A boxplot displays the significant mean differences and is produced for each paired comparison using the means of *Tukey's* test. Additionally, out of all doses that are assessed, 200 µg/mL of green trimetallic CuO/Ag/ZnO nanocomposite have the highest anti-biofilm value (Fig. 8II). On a 95% scale, the modified confidence intervals are computed using *Tukey* simultaneous tests. There are narrow statistical differences between the recorded antibiofilm percentages intervals of 150–200, and 150–250 µg/mL doses of green trimetallic CuO/Ag/ZnO nanocomposite (pass through the zero line), as seen in Fig. 8III. Due to the absence of zero line in the intervals for 200–250 µg/mL dose shows significant differences. So, the recorded MICs for all tested human pathogens range from 150 to 200 µg/mL (Table 9). An additional investigation^16^, examined the antimicrobial potential of green binary ZnO/CuO nanocomposites (irregular rod-shaped particles 7.52 nm in size) produced from *Calotropis gigantea* against drug-sensitive human pathogens (*Staphylococcus aureus* and *Escherichia coli*), multi-drug-resistant human pathogens (*Klebsiella pneumoniae*, *Pseudomonas aeruginosa*, and methicillin-resistant *S. aureus*). For S. aureus, its MICs varied between 5 and 2.5 mg/mL. Furthermore, for *E. coli, P. aeruginosa, K. pneumoniae,* and *MRSA*, the MIC values were 0.625, 0.15625, 0.625, and 0.15625 mg/mL, respectively. Therefore, our outcomes are extremely proficient, compared to earlier studies.

**Table 9:** Reduction in biofilm generation of tested human pathogens that are treated with green trimetallic CuO/Ag/ZnO nanocomposite with several doses ranging from 50 to 250 µg/mL using a biofilm inhibition assay via micro-dilution technique.

Multidrug-resistant human pathogens	Reduction in biofilm generation (% ± SD)					MICs (µg/mL)
	50 µg/mL	100 µg/mL	150 µg/mL	200 µg/mL	250 µg/mL	
*Escherichia coli*	63.45 ± 3.98d	71.55 ± 0.98c	82.36 ± 2.22ab	92.45 ± 1.41a	80.29 ± 1.85b	200
*Klebsiella pneumoniae*	67.68 ± 2.14d	73.62 ± 3.98c	89.45 ± 3.54ab	91.07 ± 1.09a	82.55 ± 0.93b	200
*Staphylococcus aureus*	68.18 ± 3.65d	75.36 ± 1.74c	95.98 ± 1.14ab	97.68 ± 1.11a	85.74 ± 3.41b	150
*Bacillus subtilis*	69.31 ± 1.98d	79.35 ± 5.54c	94.54 ± 3.54ab	98.31 ± 0.98a	82.39 ± 1.25b	150
*Candida albicans*	60.31 ± 3.54d	72.35 ± 1.09c	80.32 ± 1.12ab	90.99 ± 0.87a	82.41 ± 3.54b	200
*Candida krusei*	62.59 ± 4.58d	76.98 ± 2.33c	81.58 ± 4.56ab	89.59 ± 0.15a	83.59 ± 0.15b	200

R-sq = 86.98%, R-sq(adj) = 84.90%, and R-sq(pred) = 81.25%. The data is shown as the mean (percentage) ± standard deviation (% ± SD). Differences in the superscript letters are statistically significant at p ≤ 0.05.

**Figure 9:**
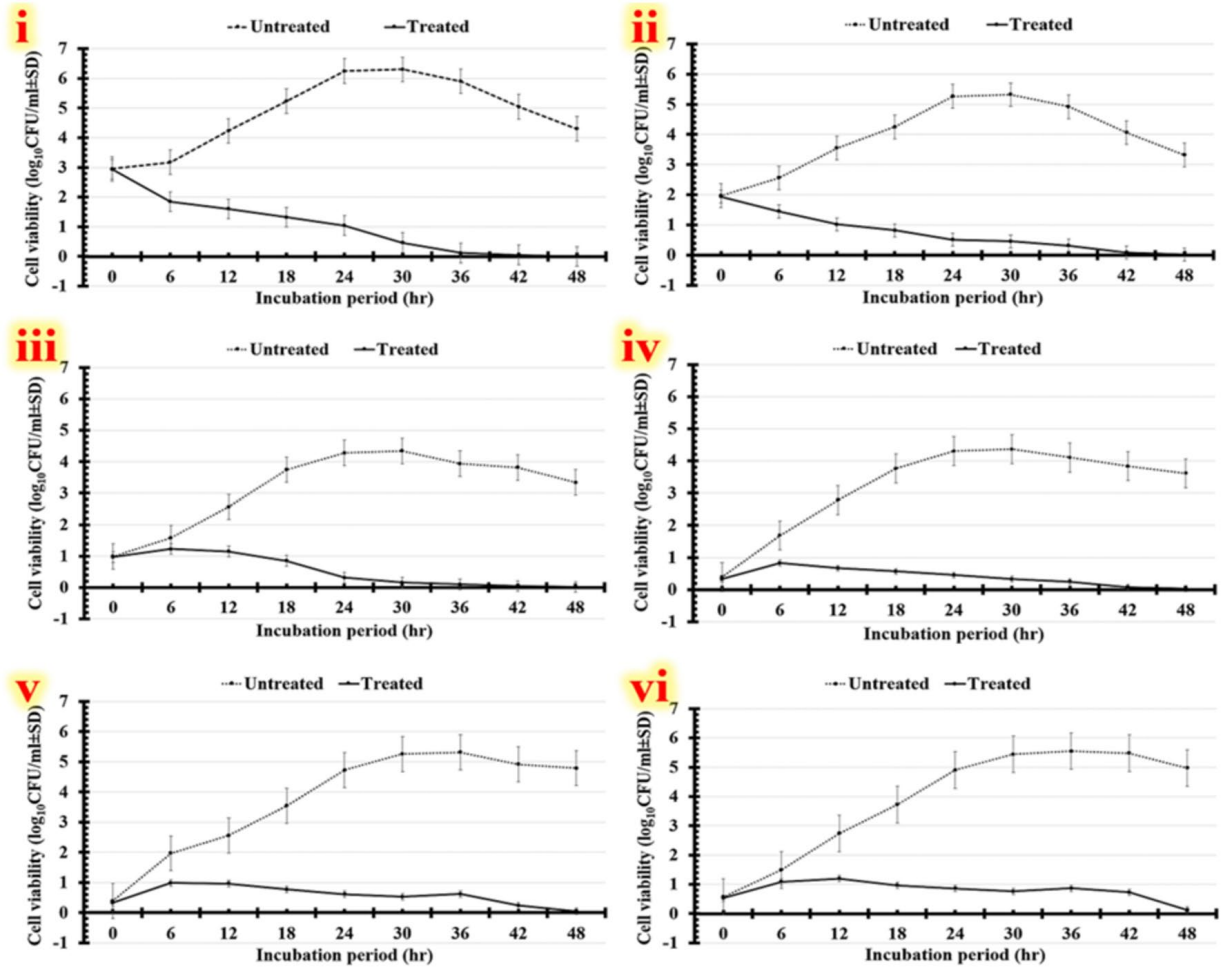
Growth rate reduction in cell viability for all multidrug-resistant human pathogens (**i**: *Escherichia coli,*** ii**: *Klebsiella pneumoniae,* **iii**: *Staphylococcus aureus,*** iv**: *Bacillus subtilis,*** v**: *Candida albicans,* and **vi**: *Candida krusei*) treated with 200 µg/mL of green trimetallic CuO/Ag/ZnO nanocomposite, as well as the untreated cells during the incubation period.

The 200 µg/mL of green trimetallic CuO/Ag/ZnO nanocomposite that demonstrates the highest degree of anti-microbial activity, is further focused for more antimicrobial exploration. The 200 µg/mL's time-kill kinetics are studied for every pathogen as part of time-kill analysis. Additionally, the log_10_ CFU/mL levels and quantitative reduction of biofilm for each examined pathogens (treated, and untreated cells) are listed in Table 10. The comparability of all studied human pathogens treated with 200 µg/mL of green trimetallic CuO/Ag/ZnO nanocomposite with the corresponding untreated cells are shown in Fig. 9. As seen, there are differences in log_10_ CFU/mL measurements within all tested human pathogens. *Gram*-positive bacteria show a significant decline in planktonic viable counts after 18 h (Fig. 9iii, iv), however *Gram*-negative bacteria (Fig. 9i, ii) and yeast cells (Fig. 9v, vi) show a similar decline after 24 h. Among the studied bacteria, *Escherichia coli*, and *Staphylococcus aureus* show the highest percentage of biofilm reduction (98.06 ± 0.93, and 97.47 ± 0.65%; respectively), and its planktonic viable counts are effectively diminished by the tested 200 µg/mL of green trimetallic CuO/Ag/ZnO nanocomposite after 36-h period. However, the planktonic viable counts of *Candida albicans* (95.42 ± 1.78%) is subsequently successfully reduced after a 36-h interval (Table 10). The time-kill assay is also utilized to ascertain the length of 200 µg/mL of green trimetallic CuO/Ag/ZnO nanocomposite that is necessary to completely eradicate the pathogens' biofilm. The biofilms of treated *Gram*-positive bacteria reveal 0% CFU/ml after 52 h; however, the biofilms are destroyed by the treated yeast cells and *Gram*-negative bacteria after 72 and 96 h; respectively. Lastly, a promising green trimetallic CuO/Ag/ZnO nanocomposite has the potential to be used as an antimicrobial substance to suppress different human pathogens that are resistant to antibiotics.

**Table 10:** Time-kill kinetics for all multidrug-resistant human pathogens treated with the 200 µg/mL of green trimetallic CuO/Ag/ZnO nanocomposite, as well as untreated cells during the incubation period.

Incubation period (h)	Multidrug-resistant human pathogens treated with 200 µg/ml of trimetallic nanocomposite (CuO/Ag/ZnO)					
	*Gram*-negative microbes					
	*Escherichia coli*			*Klebsiella pneumoniae*		
	Cell viability (log_10_CFU/ml ± SD)		Biofilm reduction (% ± SD)	Cell viability (log_10_CFU/ml ± SD)		Biofilm reduction (% ± SD)
	Untreated	Treated		Untreated	Treated	
0	2.94 ± 0.93	2.93 ± 0.95	0.33 ± 0.46	1.96 ± 0.93	1.93 ± 0.95	1.51 ± 0.67
6	3.17 ± 0.91	1.84 ± 0.98	41.90 ± 0.66	2.56 ± 0.91	1.45 ± 0.98	43.34 ± 0.12
12	4.22 ± 0.78	1.62 ± 0.56	62.10 ± 0.73	3.54 ± 0.78	1.02 ± 0.51	71.23 ± 0.35
18	5.23 ± 0.44	1.32 ± 0.19	74.72 ± 0.93	4.25 ± 0.45	0.82 ± 0.19	80.66 ± 0.25
24	6.24 ± 0.38	1.04 ± 0.39	83.31 ± 0.91	5.26 ± 0.38	0.51 ± 0.39	90.23 ± 0.52
30	6.30 ± 1.02	0.46 ± 0.12	92.67 ± 0.92	5.32 ± 0.25	0.46 ± 0.12	91.33 ± 0.42
36	5.90 ± 0.29	0.11 ± 0.39	98.06 ± 0.93	4.92 ± 0.23	0.31 ± 0.39	93.62 ± 0.37
42	5.04 ± 0.14	0.05 ± 0.01	99.82 ± 0.56	4.06 ± 0.13	0.09 ± 0.01	97.78 ± 0.11
48	4.31 ± 0.28	0.003 ± 0.05	99.93 ± 0.24	3.32 ± 1.02	0.02 ± 0.001	99.39 ± 0.19

Incubation period (h)	Multidrug-resistant human pathogens treated with 200 µg/ml of trimetallic nanocomposite (CuO/Ag/ZnO)					
	*Gram*-positive microbes					
	*Staphylococcus aureus*			*Bacillus subtilis*		
	Cell viability (log_10_CFU/ml ± SD)		Biofilm reduction (% ± SD)	Cell viability (log_10_CFU/ml ± SD)		Biofilm reduction (% ± SD)
	Untreated	Treated		Untreated	Treated	
0	0.98 ± 0.93	0.97 ± 0.01	1.95 ± 0.97	0.39 ± 0.49	0.32 ± 0.27	16.15 ± 0.52
6	1.58 ± 0.91	1.23 ± 0.54	22.19 ± 2.68	1.68 ± 0.29	0.83 ± 0.09	50.85 ± 0.84
12	2.56 ± 0.78	1.15 ± 0.36	55.21 ± 2.60	2.78 ± 1.86	0.66 ± 0.89	76.01 ± 0.91
18	3.74 ± 0.48	0.85 ± 0.15	77.30 ± 0.24	3.76 ± 0.48	0.56 ± 0.95	84.87 ± 0.34
24	4.28 ± 0.38	0.31 ± 0.39	92.67 ± 0.46	4.30 ± 1.38	0.45 ± 0.32	89.49 ± 0.57
30	4.34 ± 0.56	0.16 ± 0.13	96.28 ± 0.47	4.36 ± 0.53	0.33 ± 0.12	92.43 ± 0.29
36	3.94 ± 0.39	0.09 ± 0.01	97.47 ± 0.65	4.10 ± 0.12	0.25 ± 0.09	93.90 ± 1.68
42	3.81 ± 0.39	0.05 ± 0.11	98.68 ± 1.01	3.83 ± 1.39	0.08 ± 0.12	97.91 ± 1.36
48	3.34 ± 1.02	0.02 ± 0.03	99.40 ± 0.13	3.61 ± 0.52	0.03 ± 0.23	99.16 ± 0.41

Incubation period (h)	Multidrug-resistant human pathogens treated with 200 µg/ml of trimetallic nanocomposite (CuO/Ag/ZnO)					
	Yeast Cells					
	*Candida albicans*			*Candida krusei*		
	Cell viability (log_10_CFU/ml ± SD)		Biofilm reduction (% ± SD)	Cell viability (log_10_CFU/ml ± SD)		Biofilm reduction (% ± SD)
	Untreated	Treated		Untreated	Treated	
0	0.39 ± 1.14	0.32 ± 0.27	3.19 ± 1.89	0.57 ± 0.09	0.53 ± 0.27	2.49 ± 1.68
6	1.96 ± 0.93	0.98 ± 0.83	38.31 ± 2.17	1.49 ± 0.39	1.08 ± 0.83	14.79 ± 1.34
12	2.56 ± 0.91	0.96 ± 0.89	44.92 ± 3.86	2.74 ± 0.91	1.19 ± 0.89	41.42 ± 1.37
18	3.54 ± 0.76	0.76 ± 0.95	58.83 ± 1.37	3.72 ± 0.78	0.96 ± 0.09	56.23 ± 1.74
24	4.72 ± 0.48	0.61 ± .52	78.08 ± 1.59	4.90 ± 1.48	0.86 ± 0.15	74.27 ± 3.59
30	5.26 ± 0.38	0.53 ± 0.01	88.94 ± 1.96	5.43 ± 0.38	0.75 ± 0.93	84.37 ± 2.61
36	5.32 ± 0.25	0.62 ± 0.95	95.42 ± 1.78	5.55 ± 0.82	0.86 ± 0.25	85.32 ± 3.71
42	4.92 ± 1.27	0.23 ± 0.38	97.68 ± 2.67	5.48 ± 0.12	0.73 ± 0.28	95.45 ± 1.79
48	4.79 ± 1.39	0.05 ± 0.052	98.91 ± 0.52	4.97 ± 0.39	0.12 ± 0.52	97.48 ± 1.28

The main mechanism causing the antimicrobial effect is an interaction between the pathogenic microbes' cell wall receptors and the surface of the generated nanomaterials^33,54^. The green trimetallic CuO/Ag/ZnO nanocomposite might have direct contact with the negatively charged microbial membrane through ions released, due to surface oxidation, complicated porosity, or electrostatic interaction. According to Noohpisheh et al., there is a strong interaction between metallic silver and semiconductor zinc oxide that splits the cell membrane and increases antimicrobial activity^73^. Numerous investigations have indicated nanocomposites have superior antimicrobial properties, compared to their individual nanoparticle counterparts^81^. Based on previous studies, the green synthesized trimetallic CuO/Ag/ZnO nanocomposite damages the microbial wall, penetrates the cytoplasm, and causes cell death; because it generates superoxide and hydroxy, which alter membrane protein as well as enzyme activity^53,54,66,73^. This green synthesized trimetallic CuO/Ag/ZnO nanocomposite should therefore be used in food packaging and surgical tool coatings to prevent microbial infection and strongly inhibit the growth.

## Conclusions

In conclusion, the industrial green-synthesis of trimetallic CuO/Ag/ZnO nanocomposite was achieved by the utilization of an eco-friendly, straightforward approach that involved the extraction of sustainable resources of leaves from *Ziziphus spina christi*. Results of FTIR and phytochemical analysis showed that this extract included large concentrations of proteins, reducing sugar, anthocyanin, flavonoids, and phenolic compounds. Subsequently, the highest feasible dry weight for the green synthetic trimetallic CuO/Ag/ZnO nanocomposite was obtained by adjusting the pH of 25% diluted *Ziziphus spina-christi* extract (reductants) to 5. The precursors composed of (0.25M AgNO_3_, Cu (NO_3_)_2_.3H_2_O, and Zn (CH_3_COO)_2_.2H_2_O) were subsequently prepared at a (1:1:1) ratio and gradually titrated into this plant extract. Furthermore, distinct statistically experimental designs (*Plackett Burman* and *Taguchi* methods) were used to scaling-up the yield of green-produced trimetallic CuO/Ag/ZnO nanocomposite. Finally, the highest green synthesized trimetallic CuO/Ag/ZnO nanocomposite yield (1.65 mg/mL) might be enhanced by 1.85 and 5.7 times; respectively, by using the *Taguchi* approach in comparison to *Plackett–Burman* strategy and basal condition. In vitro, trimetallic nanocomposites have been applied to eliminate multi-drug-resistant human pathogens to determine their antimicrobial capabilities. All examined human pathogens were found to have MICs ranging from 150 to 200 µg/mL. The biofilms of treated *Gram*-positive bacteria showed 0% CFU/mL after 52 h. However, the treated *Gram*-negative bacteria and yeast cells totally eradicated the formation of biofilms after 72 and 96 h; respectively. Overall, the green synthesized trimetallic CuO/Ag/ZnO nanocomposite on a large scale has novel opportunities for the generation of antimicrobial agents that are both very stable and effective for inhibiting and preventing the microbial growth, moreover its eco-friendly generation from plant extracts.

## Data Availability

The datasets used and/or analyzed during the current study available from the corresponding authors (A.K. EL-Sawaf and E.A. Kamoun) on reasonable request.

## References

[1] Bhole, R. *et al.* Superparamagnetic spherical magnetite nanoparticles: Synthesis, characterization and catalytic potential. *Appl. Nanosci.* **13**(9), 6003–6014. 10.1007/s13204-022-02532-4 (2023). 10.1007/s13204-022-02532-4

[2] Sridevi, H., Bhat, M. R., Kumar, P. S., Kumar, N. M. & Selvaraj, R. Structural characterization of cuboidal α-Fe2O3 nanoparticles synthesized by a facile approach. *Appl. Nanosci.* **13**(8), 5605–5613. 10.1007/s13204-023-02780-y (2023). 10.1007/s13204-023-02780-y

[3] El-Moslamy, S. H., Abd-Elhamid, A. I. & El Fawal, G. Large-scale production of myco-fabricated ZnO/MnO nanocomposite using endophytic Colonstachys rosea with its antimicrobial efficacy against human pathogens. *Sci. Rep.* **14**(1), 1–20. 10.1038/s41598-024-51398-9 (2024). 38195769 10.1038/s41598-024-51398-9 PMC10776836

[4] Shet, V. B. *et al.* Cocoa pod shell mediated silver nanoparticles synthesis, characterization, and their application as nanocatalyst and antifungal agent. *Appl. Nanosci.* **13**(6), 4235–4245. 10.1007/s13204-023-02873-8 (2023). 10.1007/s13204-023-02873-8

[5] Razavi, R., Amiri, M., Alshamsi, H. A., Eslaminejad, T. & Salavati-Niasari, M. Green synthesis of Ag nanoparticles in oil-in-water nano-emulsion and evaluation of their antibacterial and cytotoxic properties as well as molecular docking. *Arab. J. Chem.* **14**(9), 103323. 10.1016/j.arabjc.2021.103323 (2021). 10.1016/j.arabjc.2021.103323

[6] Abbas, H. H., Al-Saadi, N. H. & Hassan, A. H. Green synthesis of zinc oxide-nanoparticles from Ziziphus Spina-Christi leaves extract: Characterization and their protective effects against liver disturbances in adenine-exposed male rats. *Trends Sci.* **20**, 10. 10.48048/tis.2023.6909 (2023). 10.48048/tis.2023.6909

[7] Tilahun, E., Adimasu, Y. & Dessie, Y. Biosynthesis and optimization of ZnO nanoparticles using Ocimum lamifolium leaf extract for electrochemical sensor and antibacterial activity. *ACS Omega* **8**(30), 27344–27354. 10.1021/acsomega.3c02709 (2023). 37546677 10.1021/acsomega.3c02709 PMC10399153

[8] Abdelkhalek, A. & Al-Askar, A. A. Green synthesized ZnO nanoparticles mediated by Mentha spicata extract induce plant systemic resistance against Tobacco mosaic virus. *Appl. Sci.* **10**, 15. 10.3390/app10155054 (2020). 10.3390/app10155054

[9] Stoyanova, D. *et al.* Modified approach using Mentha arvensis in the synthesis of ZnO nanoparticles—textural, structural, and photocatalytic properties. *Appl. Sci.* **12**, 3. 10.3390/app12031096 (2022). 10.3390/app12031096

[10] Ahmad, N. *et al.* Antimicrobial efficacy of Mentha piperata-derived biogenic zinc oxide nanoparticles against UTI-resistant pathogens. *Sci. Rep.* **13**(1), 1–16. 10.1038/s41598-023-41502-w (2023). 37696980 10.1038/s41598-023-41502-w PMC10495404

[11] Govindasamy, G. A., Mydin, R. B. S. M. N., Sreekantan, S. & Harun, N. H. Compositions and antimicrobial properties of binary ZnO–CuO nanocomposites encapsulated calcium and carbon from Calotropis gigantea targeted for skin pathogens. *Sci. Rep.* **11**(1), 1–14. 10.1038/s41598-020-79547-w (2021). 33420110 10.1038/s41598-020-79547-w PMC7794424

[12] Ahamad Khan, M. *et al.* Phytogenically synthesized zinc oxide nanoparticles (ZnO-NPs) potentially inhibit the bacterial pathogens: In vitro studies. *Toxics* **11**(5), 1–23. 10.3390/toxics11050452 (2023). 10.3390/toxics11050452 PMC10221642 37235266

[13] Mahmoud, A. E. D., El-Maghrabi, N., Hosny, M. & Fawzy, M. Biogenic synthesis of reduced graphene oxide from Ziziphus spina-christi (Christ’s thorn jujube) extracts for catalytic, antimicrobial, and antioxidant potentialities. *Environ. Sci. Pollut. Res.* **29**(59), 89772–89787. 10.1007/s11356-022-21871-x (2022). 10.1007/s11356-022-21871-x PMC9671977 35859234

[14] Dhivya, A., Yadav, R. & Powrnika, S. Green synthesis of selenium doped zinc oxide nanoparticles using Mangifera indica leaf extract and its photodegradation and antibacterial activities. *J. Nanosci. Technol.* **5**(3), 741–744. 10.30799/jnst.s08.19050310 (2019). 10.30799/jnst.s08.19050310

[15] Kunwar, S. *et al.* Bio-fabrication of Cu/Ag/Zn nanoparticles and their antioxidant and dye degradation activities. *Catalysts* **13**(5), 1–19. 10.3390/catal13050891 (2023). 10.3390/catal13050891

[16] Sabouri, Z. *et al.* Phytoextract-mediated synthesis of Ag-doped ZnO–MgO–CaO nanocomposite using Ocimum Basilicum L seeds extract as a highly efficient photocatalyst and evaluation of their biological effects. *Ceram. Int.* **49**(12), 20989–20997. 10.1016/j.ceramint.2023.03.234 (2023). 10.1016/j.ceramint.2023.03.234

[17] Abbas, S. *et al.* Dual-functional green facile CuO/MgO nanosheets composite as an efficient antimicrobial agent and photocatalyst. *Arab. J. Sci. Eng.* **1**, 1. 10.1007/s13369-021-05741-1 (2021). 10.1007/s13369-021-05741-1

[18] Moeen, M. *et al.* Green synthesis, characterization and sorption efficiency of MnO2 nanoparticles and MnO2@waste eggshell nanocomposite. *J. Taibah Univ. Sci.* **16**(1), 1075–1095. 10.1080/16583655.2022.2139483 (2022). 10.1080/16583655.2022.2139483

[19] Abdelaziz, A. M. *et al.* Ziziphus spina-christi extract-stabilized novel silver nanoparticle synthesis for combating Fusarium oxysporum-causing pepper wilt disease: in vitro and in vivo studies. *Arch. Microbiol.* **205**(2), 1–17. 10.1007/s00203-023-03400-7 (2023). 10.1007/s00203-023-03400-7 36670250

[20] Erol, I., Sivrier, M., Cigerci, I. H., Özkara, A. & Akyıl, D. ZnO-containing nanocomposites produced from Mentha pulegium L. of a new HEMA-based methacrylate copolymer: Improvement the thermal and antimicrobial effect. *J. Polym. Res.* **30**, 3. 10.1007/s10965-023-03461-8 (2023). 10.1007/s10965-023-03461-8

[21] Suresh, J., Ragunath, L. & Hong, S. I. Biosynthesis of mixed nanocrystalline Zn-Mg-Cu oxide nanocomposites and their antimicrobial behavior. *Adv. Nat. Sci. Nanosci. Nanotechnol.* **10**, 4. 10.1088/2043-6254/ab52f5 (2019). 10.1088/2043-6254/ab52f5

[22] Baig, A. U. *et al.* Facile green synthesis of silver doped zno nanoparticles using tridax procumbens leaf extract and their evaluation of antibacterial activity. *J. Water Environ. Nanotechnol.* **5**(4), 307–320. 10.22090/jwent.2020.04.002 (2020). 10.22090/jwent.2020.04.002

[23] Panchal, P. *et al.* Eco-friendly synthesis of Ag-doped ZnO/MgO as a potential photocatalyst for antimicrobial and dye degradation applications. *Coord. Chem. Rev.* **493**, 215283. 10.1016/j.ccr.2023.215283 (2023). 10.1016/j.ccr.2023.215283

[24] Vijayaram, S. *et al.* Applications of green synthesized metal nanoparticles—a review. *Biol. Trace Elem. Res.* **1**, 1. 10.1007/s12011-023-03645-9 (2023). 10.1007/s12011-023-03645-9 PMC10097525 37046039

[25] Sabouri, Z., Kazemi, M. & Sabouri, M. Biosynthesis of Ag doped MgO-NiO-ZnO nanocomposite with Ocimum Basilicum L extract and assessment of their biological and photocatalytic applications. *J. Mol. Struct.* **1306**, 137895. 10.1016/j.molstruc.2024.137895 (2024). 10.1016/j.molstruc.2024.137895

[26] Makauki, E., Mtavangu, S. G., Basu, O. D., Rwiza, M. & Machunda, R. Facile biosynthesis of Ag–ZnO nanocomposites using Launaea cornuta leaf extract and their antimicrobial activity. *Discov. Nano* **18**, 1. 10.1186/s11671-023-03925-2 (2023). 37975945 10.1186/s11671-023-03925-2 PMC10656379

[27] Jakinala, P. *et al.* Green synthesis of ZnO-Ag nanocomposite using Stenotaphrum secundatum grass extract: Antibacterial activity and anticancer effect in oral squamous cell carcinoma CAL 27 cells. *Inorg. Chem. Commun.* **152**, 110735. 10.1016/j.inoche.2023.110735 (2023). 10.1016/j.inoche.2023.110735

[28] Jalil, P. J., Shnawa, B. H., Hamad, S. M., Hamad, B. S. & Ahmed, M. H. The efficiency of fabricated Ag/ZnO nanocomposite using Ruta chalepensis L. leaf extract as a potent protoscolicidal and anti-hydatid cysts agent. *J. Biomater. Appl.* **38**(5), 629–645. 10.1177/08853282231207236 (2023). 37844268 10.1177/08853282231207236

[29] Pompapathi, K. *et al.* Visible-light-driven mentha spicata L.-mediated Ag-doped Bi2Zr2O7 nanocomposite for enhanced degradation of organic pollutants, electrochemical sensing, and antibacterial applications. *ACS Environ.* **1**, 1. 10.1021/acsenvironau.3c00057 (2023). 10.1021/acsenvironau.3c00057 PMC10958660 38525021

[30] Mohanaparameswari, S. *et al.* Investigation of structural properties and antibacterial activity of AgO nanoparticle extract from Solanum nigrum/Mentha leaf extracts by green synthesis method. *Green Process. Synth.* **12**(1), 1–17. 10.1515/gps-2023-0080 (2023). 10.1515/gps-2023-0080

[31] El-Shahir, A. A., El-Wakil, D. A., Latef, A. A. H. A. & Youssef, N. H. Bioactive compounds and antifungal activity of leaves and fruits methanolic extracts of Ziziphus spina-christi L. *Plants* **11**(6), 1–18. 10.3390/plants11060746 (2022). 10.3390/plants11060746 PMC8955299 35336628

[32] Souri, M. & Shakeri, A. Optimization of total phenol and tannin content and biological activity of Dittrichia graveolens (L.) GREUTER. *Curr. Bioact. Compd.* **16**(2), 124–132. 10.2174/1573407214666180730110830 (2018). 10.2174/1573407214666180730110830

[33] Seddiek, A. S., Hamad, G. M., Zeitoun, A. A., Zeitoun, M. A. M. & Ali, S. Antimicrobial and antioxidant activity of some plant extracts against different food spoilage and pathogenic microbes. *Eur. J. Nutr. Food Saf.* **1**, 1–12. 10.9734/ejnfs/2020/v12i1130312 (2020). 10.9734/ejnfs/2020/v12i1130312

[34] Verma, A., Kumar, R., Kumari, S., Lata, S., & Zinzala, V. Synthesized nano ZnO and its comparative effects with ZnO and heptahydrate ZnSO4 on sweet corn (Zea mays L. saccharata). *Pharma Innov. J.* **10**(10), 1–7 [Online]. Available: http://www.thepharmajournal.com (2021).

[35] Adak, T. *et al.* Green silver nano-particles: Synthesis using rice leaf extract, characterization, efficacy, and non-target effects. *Environ. Sci. Pollut. Res.* **1**, 4452–4462. 10.1007/s11356-020-10601-w (2020). 10.1007/s11356-020-10601-w 32944855

[36] El-Moslamy, S. H., Yahia, I. S., Zahran, H. Y. & Kamoun, E. A. Novel biosynthesis of MnO NPs using Mycoendophyte: Industrial bioprocessing strategies and scaling-up production with its evaluation as anti-phytopathogenic agents. *Sci. Rep.* **13**(1), 1–21. 10.1038/s41598-023-28749-z (2023). 36739323 10.1038/s41598-023-28749-z PMC9899258

[37] El-Moslamy, S. H., El-Morsy, E. S. M., Mohaisen, M. T., Rezk, A. H. & Abdel-Fattah, Y. R. Industrial bioprocessing strategies for cultivation of local Streptomyces violaceoruber strain SYA3 to fabricate nano-ZnO as anti-phytopathogens agent. *J. Pure Appl. Microbiol.* **12**(3), 1133–1145. 10.22207/JPAM.12.3.12 (2018). 10.22207/JPAM.12.3.12

[38] El-Moslamy, S. H. Bioprocessing strategies for cost-effective large-scale biogenic synthesis of nano-MgO from endophytic Streptomyces coelicolor strain E72 as an anti-multidrug-resistant pathogens agent. *Sci. Rep.* **8**(1), 1–22. 10.1038/s41598-018-22134-x (2018). 29491452 10.1038/s41598-018-22134-x PMC5830579

[39] CLSI. Methods for antimicrobial broth dilution and disk diffusion susceptibility testing of bacteria isolated from aquatic animals. In *VET03-Clinical and Laboratory Standards Institute*, vol. VET03, 2020, p. 122. Accessed: Oct. 19, 2022. [Online]. Available: www.clsi.org.

[40] El-Moslamy, S. H., Elnouby, M. S., Rezk, A. H. & El-Fakharany, E. M. Scaling-up strategies for controllable biosynthetic ZnO NPs using cell free-extract of endophytic Streptomyces albus: characterization, statistical optimization, and biomedical activities evaluation. *Sci. Rep.* **13**(1), 1–22. 10.1038/s41598-023-29757-9 (2023). 36823304 10.1038/s41598-023-29757-9 PMC9950444

[41] Kakian, F., Mirzaei, E., Moattari, A., Takallu, S. & Bazargani, A. Determining the cytotoxicity of the Minimum Inhibitory Concentration (MIC) of silver and zinc oxide nanoparticles in ESBL and carbapenemase producing Proteus mirabilis isolated from clinical samples in Shiraz, Southwest Iran. *BMC Res. Notes* **17**(1), 1–6. 10.1186/s13104-023-06402-2 (2024). 38287416 10.1186/s13104-023-06402-2 PMC10826267

[42] Titus, D., James Jebaseelan Samuel, E., & Roopan, S. M. Nanoparticle characterization techniques. In *Green Synthesis, Characterization and Applications of Nanoparticles*, pp. 303–319. (Elsevier, 2019). 10.1016/b978-0-08-102579-6.00012-5.

[43] Betiha, M. A. *et al.* A review on different plants extract mediated silver nanoparticles: Preparation, antimicrobials, and antioxidant. *Egypt. J. Chem.* **65**(5), 575–589. 10.21608/ejchem.2021.99747.4637 (2022). 10.21608/ejchem.2021.99747.4637

[44] Arvanagh, F. M. *et al.* Anti-inflammatory and collagenation effects of zinc oxide-based nanocomposites biosynthesised with Mentha longifolia leaf extract. *J. Wound Care* **32**(1), 44–54. 10.12968/jowc.2023.32.1.44 (2023). 36630114 10.12968/jowc.2023.32.1.44

[45] Consolo, V. F., Torres-Nicolini, A. & Alvarez, V. A. Mycosinthetized Ag, CuO and ZnO nanoparticles from a promising Trichoderma harzianum strain and their antifungal potential against important phytopathogens. *Sci. Rep.* **10**(1), 1–10. 10.1038/s41598-020-77294-6 (2020). 33235262 10.1038/s41598-020-77294-6 PMC7687894

[46] Shinde, R. S. *et al.* Design, fabrication, antitubercular, antibacterial, antifungal and antioxidant study of silver doped ZnO and CuO nano candidates: A comparative pharmacological study. *Curr. Res. Green Sustain. Chem.* **4**, 100138. 10.1016/j.crgsc.2021.100138 (2021). 10.1016/j.crgsc.2021.100138

[47] Singh, J. *et al.* ‘Green’ synthesis of metals and their oxide nanoparticles: Applications for environmental remediation. *J. Nanobiotechnol.* **16**, 1. 10.1186/s12951-018-0408-4 (2018). 10.1186/s12951-018-0408-4 PMC6206834 30373622

[48] Jubair, N., Rajagopal, M., Chinnappan, S., Abdullah, N. B. & Fatima, A. Review on the antibacterial mechanism of plant-derived compounds against multidrug-resistant bacteria (MDR). *Evid. Based Complement. Altern. Med.* **1**, 1. 10.1155/2021/3663315 (2021). 10.1155/2021/3663315 PMC8384518 34447454

[49] Khosravi, R. *et al.* Facile green synthesis of zinc oxide nanoparticles using Thymus vulgaris extract, characterization, and mechanism of chromium photocatalytic reduction. *Mater. Res. Express* **6**(11), 115093. 10.1088/2053-1591/ab4ae7 (2019). 10.1088/2053-1591/ab4ae7

[50] Pekdemir, M. E., Pekdemir, S., İnci, Ş, Kırbağ, S. & Çiftci, M. Thermal, magnetic properties and antimicrobial effects of magnetic iron oxide nanoparticles treated with polygonum cognatum. *Iran. J. Sci. Technol. Trans. A Sci.* **45**(5), 1579–1586. 10.1007/s40995-021-01167-4 (2021). 10.1007/s40995-021-01167-4

[51] Tabrizi Hafez Moghaddas, S. S., Samareh Moosavi, S. & Kazemi Oskuee, R. Green synthesis of calcium oxide nanoparticles in Linum usitatissimum extract and investigation of their photocatalytic and cytotoxicity effects. *Biomass Convers. Biorefinery* **14**(4), 5125–5134. 10.1007/s13399-022-02643-6 (2024). 10.1007/s13399-022-02643-6

[52] Pujar, M., Kumar, K. & Kumar, P. IRJET- Recent Trends in Green Synthesis of ZnO Nanomaterials Using Plant Extracts. *Int. Res. J. Eng. Technol.* **08**, 152–163 (2021).

[53] Azad, A., Zafar, H., Raza, F. & Sulaiman, M. Factors influencing the green synthesis of metallic nanoparticles using plant extracts: A comprehensive review. *Pharm. Front.* **05**(03), e117–e131. 10.1055/s-0043-1774289 (2023). 10.1055/s-0043-1774289

[54] Nzilu, D. M. *et al.* Green synthesis of copper oxide nanoparticles and its efficiency in degradation of rifampicin antibiotic. *Sci. Rep.* **13**(1), 1–18. 10.1038/s41598-023-41119-z (2023). 37640783 10.1038/s41598-023-41119-z PMC10462644

[55] Bin Chan, Y. *et al.* Impact of diverse parameters on the physicochemical characteristics of green-synthesized zinc oxide-copper oxide nanocomposites derived from an aqueous extract of garcinia mangostana L. Leaf. *Materials* **16**(15), 1. 10.3390/ma16155421 (2023). 10.3390/ma16155421 PMC10419950 37570124

[56] Mohamed, A. A., Abu-Elghait, M., Ahmed, N. E. & Salem, S. S. Correction to: Eco-friendly mycogenic synthesis of ZnO and CuO nanoparticles for in vitro antibacterial, antibiofilm and antifungal applications (biological trace element research, (2021), 199, 7, (2788–2799), DOI: 10.1007/s12011-020-02369-4). *Biol. Trace Elem. Res.* **199**(7), 2800–2801. 10.1007/s12011-020-02391-6 (2021). 32974847 10.1007/s12011-020-02391-6

[57] Asamoah, R. B., *et al.* A comparative study of antibacterial activity of CuO/Ag and ZnO/Ag nanocomposites. *Adv. Mater. Sci. Eng.*10.1155/2020/7814324 (2020).

[58] El Moslamy, S. H. & Kabeil, S. S. A. Bioprocess development for chlorella vulgaris cultivation and biosynthesis of anti-phytopathogens silver nanoparticles. *J. Nanomater. Mol. Nanotechnol.* **5**(1), 1. 10.4172/2324-8777.1000177 (2016). 10.4172/2324-8777.1000177

[59] El-Moslamy, S. H., & Abdel-Fattah, Y. R. Statistical bioprocessing strategy for cellulases production by endophytic Trichoderma harzianum utilizing lignocellulosic wastes. *Biosci. Res.* **15**(3), 1852–1866. Available: www.isisn.org (2018).

[60] El-Moslamy, S. H., Elnouby, M. S., Rezk, A. H. & El-Fakharany, E. M. Scaling-up strategies for controllable biosynthetic ZnO NPs using cell free-extract of endophytic Streptomyces albus: characterization, statistical optimization, and biomedical activities evaluation. *Sci. Rep.* **13**, 1. 10.1038/s41598-023-29757-9 (2023). 36823304 10.1038/s41598-023-29757-9 PMC9950444

[61] S. H. EL-Moslamy, A. H. Rezk, M. F. Elkady, and H. Shokry Hassan,. Semi-industrial bio-fabrication of ZnO/MnO2 nanocomposite using endophytic streptomyces coelicolor: Characterization, statistical design, exponential pulse fed-batch fermentation, and its antimicrobial application. *Arab. J. Sci. Eng.* **49**, 9067–9088. 10.1007/s13369-024-08709-z (2024). 10.1007/s13369-024-08709-z

[62] El-Moslamy, S. H., Yahia, I. S., Zahran, H. Y. & Kamoun, E. A. Novel biosynthesis of MnO NPs using Mycoendophyte: Industrial bioprocessing strategies, characterization, scaling-up production, and its evaluation as anti-phytopathogenic agents. *Sci. Rep.* **1**, 1–21. 10.1038/s41598-023-28749-z (2022). 10.1038/s41598-023-28749-z PMC9899258 36739323

[63] Sabouri, Z., Sammak, S., Sabouri, S., Sadat, S., & Hafez, T. Green synthesis of Ag-Se doped ZnO-Co 3 O 4 -NiO fivenary nanocomposite using poly anionic cellulose and evaluation of their anticancer and photocatalyst applications. **8**, 164–176 (2024).

[64] Mahmood, K. *et al.* Green synthesis of Ag@CdO nanocomposite and their application towards brilliant green dye degradation from wastewater. *J. Nanostructure Chem.* **12**(3), 329–341. 10.1007/s40097-021-00418-5 (2022). 10.1007/s40097-021-00418-5

[65] Karvekar, O. S. *et al.* Biogenic synthesis of silver anchored ZnO nanorods as nano catalyst for organic transformation reactions and dye degradation. *Appl. Nanosci.* **12**(7), 2207–2226. 10.1007/s13204-022-02470-1 (2022). 35466324 10.1007/s13204-022-02470-1 PMC9019544

[66] Vinayagam, R. *et al.* Green synthesized hydroxyapatite nanoadsorbent for the adsorptive removal of AB113 dye for environmental applications. *Environ. Res.* **212**, 1. 10.1016/j.envres.2022.113274 (2022). 10.1016/j.envres.2022.113274 35461848

[67] Varadavenkatesan, T., Pai, S., Vinayagam, R. & Selvaraj, R. Characterization of silver nano-spheres synthesized using the extract of Arachis hypogaea nuts and their catalytic potential to degrade dyes. *Mater. Chem. Phys.* **272**, 1. 10.1016/j.matchemphys.2021.125017 (2021). 10.1016/j.matchemphys.2021.125017

[68] Abd El-Mohdy, H. L. & Aly, H. M. Characterization, properties and antimicrobial activity of radiation induced phosphorus-containing PVA hydrogels. *Arab. J. Sci. Eng.* **48**(1), 341–351. 10.1007/s13369-022-07031-w (2023). 10.1007/s13369-022-07031-w

[69] Kumar, Y. *et al.* Development and characterization of defatted pumpkin seed meal and halloysite nanoclay composite films for food packaging. *Packag. Technol. Sci.* **36**(8), 715–727. 10.1002/pts.2751 (2023). 10.1002/pts.2751

[70] Fekri, R., Mirbagheri, S. A., Fataei, E., Ebrahimzadeh-Rajaei, G. & Taghavi, L. Green synthesis of CuO nanoparticles using Peganum harmala extract for photocatalytic and sonocatalytic degradation of reactive dye and organic compounds. *Main Gr. Chem.* **21**(4), 975–996. 10.3233/MGC-220045 (2022). 10.3233/MGC-220045

[71] Al-Qadsy, I. *et al.* Antimicrobial activity of novel Ni(II) and Zn(II) Complexes with (E)-2-((5-Bromothiazol-2-yl)imino)methyl)phenol Ligand: Synthesis, characterization and molecular docking studies. *Antibiotics* **12**, 11. 10.3390/antibiotics12111634 (2023). 10.3390/antibiotics12111634 PMC10669075 37998835

[72] Aasy, N. K. A. *et al.* Concurrent tissue engineering for wound healing in diabetic rats utilizing dual actions of green synthesized CuO NPs prepared from two plants grown in Egypt. *Int. J. Nanomed.* **18**(April), 1927–1947. 10.2147/IJN.S397045 (2023). 10.2147/IJN.S397045 PMC10103783 37064292

[73] Yang, Y., Waterhouse, G. I. N., Chen, Y., Sun-Waterhouse, D. & Li, D. Microbial-enabled green biosynthesis of nanomaterials: Current status and future prospects. *Biotechnol. Adv.* **55**, 107914. 10.1016/j.biotechadv.2022.107914 (2022). 35085761 10.1016/j.biotechadv.2022.107914

[74] Pakseresht, S. *et al.* Review—nanomaterials green synthesis for high-performance secondary rechargeable batteries: Approaches, challenges, and perspectives. *J. Electrochem. Soc.* **169**(1), 010534. 10.1149/1945-7111/ac4843 (2022). 10.1149/1945-7111/ac4843

[75] El-Belely, E. F. *et al.* Green synthesis of zinc oxide nanoparticles (Zno-nps) using arthrospira platensis (class: Cyanophyceae) and evaluation of their biomedical activities. *Nanomaterials* **11**(1), 1–18. 10.3390/nano11010095 (2021). 10.3390/nano11010095 PMC7823323 33406606

[76] Jamdagni, P., Rana, J. S., Khatri, P. & Nehra, K. Comparative account of antifungal activity of green and chemically synthesized Zinc Oxide nanoparticles in combination with agricultural fungicides. *Int. J. Nano Dimens.* **9**(2), 198–208 (2018).

[77] Sharma, R. *et al.* Statistical optimization of process parameters for improvement of phycobiliproteins (PBPs) yield using ultrasound-assisted extraction and its kinetic study. *Ultrason. Sonochem.* **60**, 104762. 10.1016/j.ultsonch.2019.104762 (2020). 31546084 10.1016/j.ultsonch.2019.104762

[78] Rizal, S. *et al.* Preparation of palm oil ash nanoparticles: Taguchi optimization method by particle size distribution and morphological studies. *Appl. Sci.* **10**(3), 985. 10.3390/app10030985 (2020). 10.3390/app10030985

[79] Chen, H. *et al.* Characterization and source identification of antibiotic resistance genes in the sediments of an interconnected river-lake system. *Environ. Int.* **137**, 105538. 10.1016/j.envint.2020.105538 (2020). 32028174 10.1016/j.envint.2020.105538

[80] Haseena, A. *et al.* Synthesis of ribose-coated copper-based metal-organic framework for enhanced antibacterial potential of chloramphenicol against multi-drug resistant bacteria. *Antibiotics* **10**, 12. 10.3390/antibiotics10121469 (2021). 10.3390/antibiotics10121469 PMC8698127 34943681

[81] Noohpisheh, Z., Amiri, H., Farhadi, S., Mohammadi-gholami, A. Green synthesis of Ag-ZnO nanocomposites using Trigonella foenum-graecum leaf extract and their antibacterial, antifungal, antioxidant and photocatalytic properties. *Spectrochim. Acta Part A Mol. Biomol. Spectrosc.* **240**, 118595. 10.1016/j.saa.2020.118595 (2020). 10.1016/j.saa.2020.118595 32599480

[82] Javed, B., Nadhman, A., Razzaq, A. & Mashwani, Z. U. R. One-pot phytosynthesis of nano-silver from *Mentha longifolia* L.: Their characterization and evaluation of photodynamic potential. *Mater. Res. Express* **7**, 5. 10.1088/2053-1591/ab903b (2020). 10.1088/2053-1591/ab903b

[83] Krishnaraj, C., Ji, B. J., Harper, S. L. & Il Yun, S. Plant extract-mediated biogenic synthesis of silver, manganese dioxide, silver-doped manganese dioxide nanoparticles and their antibacterial activity against food- and water-borne pathogens. *Bioprocess Biosyst. Eng.* **39**(5), 759–772. 10.1007/s00449-016-1556-2 (2016). 26857369 10.1007/s00449-016-1556-2

[84] Said, A., Abu-Elghait, M., Atta, H. M., & Salem, S. S. Antibacterial activity of green synthesized silver nanoparticles using Lawsonia inermis against common pathogens from urinary tract infection. *Appl. Biochem. Biotechnol.* 0123456789. 10.1007/s12010-023-04482-1 (2023). 10.1007/s12010-023-04482-1 PMC10794286 37099124

[85] Lashin, I., Hasanin, M., Hassan, S. A. M. & Hashem, A. H. Green biosynthesis of zinc and selenium oxide nanoparticles using callus extract of Ziziphus spina-christi: Characterization, antimicrobial, and antioxidant activity. *Biomass Convers. Biorefinery* **13**(11), 10133–10146. 10.1007/s13399-021-01873-4 (2023). 10.1007/s13399-021-01873-4

